# Supplementary material for: Structural dynamics in proteins induced by and probed with X-ray free-electron laser pulses
Source: Nat Commun. 2020 Apr 14;11:1814. doi: 10.1038/s41467-020-15610-4 (PMC7156470; doi:10.1038/s41467-020-15610-4)
Supplement: Supplementary file 1 — Supplementary Information [file 41467_2020_15610_MOESM1_ESM.pdf]

Supplementary Information:

Structural dynamics in proteins induced by and probed with X-ray free-electron laser pulses

Karol Nass, Alexander Gorel et al.

Supplementary Note 1

Superposition of the individual disulphide bridges in a common coordinate system

To superimpose the disulphide bridges within a common coordinate system, we introduce local coordinates. A right-handed orthogonal coordinate system is delineated by first defining two characteristic points in the S-S bridge, namely the midpoint P_m and the intersection point P_i. The midpoint is located halfway between the sulphur Sγ atoms. A plane is passed, normal to the S-S connecting line, through the midpoint. The intersection of this plane with the line connecting the respective Cβ atoms defines the intersection point (Supplementary Fig. 9a). Mathematically a orthogonal coordinate system is then defined as follows, using the nomenclature resented in Supplementary Fig. 9b. The coordinates of the Sγ atoms of the disulphide bridges are P_2, P_3 and the coordinates of the Cβ atoms are P_1,P_4, respectively.

The midpoint is calculated as $P_m = \frac{P_2+P_3}{2}$ (1)

We define the normal vector $\vec{n}_{23} = \frac{P_3-P_2}{|P_3-P_2|}$ (2)

and the moving point $P_{mov} = P_1 + t \cdot (P_4 - P_1)$. (3)

To find the point on the line between P_1 and P_4 that lies in the plane normal to and passing through the midpoint P_m it is required that the distance between the moving point and the plane

$d = |P_{mov} - P_i|$ becomes zero, corresponding to

$$d = (P_1 + t_{sect} \cdot (P_4 - P_1) - P_m) \cdot \vec{n}_{23} = 0 \quad (4)$$

which occurs when it holds that $t_{sect} = \frac{(P_m-P_1)\cdot\vec{n}_{23}}{(P_4-P_1)\cdot\vec{n}_{23}}$ (5)

with the intersection point $P_i = P_1 + \frac{(P_m-P_1)\cdot\vec{n}_{23}}{(P_4-P_1)\cdot\vec{n}_{23}} \cdot (P_4 - P_1)$. (6)

The local coordinate system is defined using the normal vector $\vec{n}_{23} = \frac{P_3-P_2}{|P_3-P_2|}$ (7)

and the unit vector $\vec{o}_{23} = \frac{P_m-P_i}{|P_m-P_i|}$ (8)

anchored by four characteristic points

$$P_m, P_x = P_m + \vec{n}_{23}, P_y = P_m + \vec{o}_{23} \times \vec{n}_{23} \text{ and } P_z = P_m + \vec{o}_{23}. \quad (9)$$

To find the affine coordinate transformation the

start matrix $M_{start} = \begin{pmatrix} P_x & P_y & P_z & P_m \\ 1 & 1 & 1 & 1 \end{pmatrix}$ (10)

and the target matrix $M_{target} = \begin{pmatrix} 1 & 0 & 0 & 0 \\ 0 & 1 & 0 & 0 \\ 0 & 0 & 1 & 0 \\ 1 & 1 & 1 & 1 \end{pmatrix}$ (11)

is used, yielding the affine transform matrix $M_{affine} \cdot M_{start} = M_{target}$. (12)

Then the pseudoinverse matrix is calculated and the system of equations is solved for the affine transform $M_{affine} = \left(M_{target} \cdot {M_{start}}^{T}\right) \cdot \left(M_{start} \cdot {M_{start}}^{T}\right)^{-1}$. (13)

The local coordinate systems of the disulphide bridges was used to transform the coordinates of the Sγ and Cβ atoms at various time points into one uniform coordinate system. Then the X-Y and X-Z projections in the uniform coordinate system were calculated to facilitate interpretation of the structural changes (Supplementary Fig. 11).

Supplementary Note 2

2.0 Experimental results

2.1 Overall data quality

Global radiation damage manifests itself among other things in the overall scaling behaviour of the diffracted intensities with resolution in a dose and dose-rate dependent manner[1,2]. This becomes difficult to analyse if the experiment is performed with crystals that are significantly larger than the X-ray beam focus. In that situation, only small parts of the crystals are exposed to the highly intense part of the beam while significantly larger parts of the crystals are grazed by the lower-intensity "halo" around the focus. This results in different apparent degrees of damage for crystals of varying size, at different parts of the crystal. This was the case for our previous experiment using the strongly diffracting ferredoxin crystals, where even very weak parts of the X-ray beam likely gave rise to appreciable diffraction signal[3]. To avoid this complication, the current experiment employed nanocrystals of lysozyme and thaumatin similar in size to the X-ray beam focus.

2.2 Disulphide bonds and methionine residues

The averaged changes in S-S and Cβ-Sγ distances with pulse separation time delay are shown in Figure 1 for thaumatin and lysozyme.Gd, respectively. Individual changes in these distances are

shown in Supplementary Fig. 8. The sulphur displacements in thaumatin and lysozyme.Gd differ significantly for the nominally 20 and 40 fs time delays. Moreover, the displacement for the 35 fs and 37 fs time delays (nominally 20 and 40 fs) for lysozyme.Gd also differ significantly, which is not physically feasible. Both effects are likely due to differences in accelerator operation that altered beam attributes, such as pointing, and thereby the intensity at the sample interaction region. Accordingly it is unclear whether the plateau and corresponding dip in the S-S and Sγ-Cβ displacement plot at ~40 fs time delay are real or only artefacts (Supplementary Fig. 8c). Apart from these time delay values the magnitude and kinetics of the average S-S bond elongation and ultimately rupture are very similar for lysozyme.Gd and thaumatin despite their different secondary and three-dimensional structures and absorbed dose, implying that the displacement is caused by local charging. The sulphur atoms displace on average at a speed of roughly 1000 m s^{-1} (1 Å in 100 fs). The integrated electron density of the sulphur atoms decreases with time (data not shown), due to ionization or disorder or both.

The displacements of the sulphur ions in the individual S-S bonds vary (Supplementary Fig. 8). To facilitate the comparison of the sulphur displacements, we superimposed all disulphide bridges within a common coordinate system (see Supplementary Note 1). In contrast to the rather uniform increase in scalar values of the sulphur ion displacements with pump-probe delay (Supplementary Fig. 8), the superimposed trajectories of the sulphur ions are asymmetric and differ (Supplementary Fig. 10). In lysozyme.Gd the trajectories of the sulphur ions of Cys6, 30, 76, 80, 94 change direction for the last two time delays, which may be due to steric constraints. Since the trajectories of the moving sulphur ions remain close to the X-Y plane of the local coordinate system (top view of the S-S-bond, see Supplementary Fig. 9a), we calculated the intersections of spheres (1.8 Å radius, the van der Waals radius of sulphur (C, N, O are slightly smaller)) located at the positions of the sulphurs and of atoms of neighbouring residues, respectively, with the X-Y plane. These plots show the deviations of the trajectories to correlate with closeness to adjacent residues (Supplementary Fig. 12). Conversely, if there is space, the sulphur ions move further into protein voids (e.g. Cys64, 127 in lysozyme.Gd). However, the trajectories of the sulphurs of Cys6 and Cys94, which point towards the solvent, decelerate to a halt. The findings are similar for thaumatin (Supplementary Fig. 10,11). The disulphide bridge between Cys159 and Cys164 was excluded from the analysis since it has two alternative conformations preventing accurate refinement. Inspection of the trajectories of the sulphur ions within the common coordinate system, along with

the overlapping sphere analysis for steric constraints, shows the sulphur ions of Cys56, 66, 126 and 193 to move within the protein voids and the sulphur of Cys177 to collide with the carbonyl oxygen of Leu176, resulting in significant deviation of the trajectory, whereas the trajectories of the sulphur ions of Cys149, 204 carry the ions into the solvent and so to a halt (Supplementary Fig. 10).

Interestingly, in thaumatin the trajectories can be divided into two groups (Supplementary Fig. 10), one with almost identical trajectories (Cys204, 66, 77, 193, 145, 158, with the exception of Cys177) and the other with divergent trajectories (Cys9, 56, 71, 121, 126, 134, 149). This behaviour correlates with the spread of displacements of Cβ to which the sulphurs were originally bound (Supplementary Fig. 12).

To test whether this observation is due to model fitting or refinement, we calculated the projection of disulphide bridges from 38 high-resolution (0.6-1.2 Å resolution) thaumatin structures of identical sequence as deposited in the Protein Data Bank. They show the same feature of a larger spatial spread of the Cβ atoms on the "backside" of the projection along the Y-axis (Supplementary Fig. 9a) due to a symmetry break between Cβ-Sγ and Sγ-Cβ. This feature can be related to the mechanism of disulphide bond formation in folding proteins. In addition, the angular trajectory of the sulphurs implies that the sulphur ions orbit around the Cβ atoms (to which they are possibly still bound) while repelling each other.

A disulphide bond is defined by six atoms of the two cysteine residues and characterized by the five χ-angles around the corresponding bonds (Supplementary Fig. 6). Disulphide bonds in proteins differ in their local environment, the secondary structure of the constituting cysteine residues, their solvent accessibility and their geometry[4]. Supplementary Table 3 lists these parameters as well as the classification of the disulphide conformations. We conjectured that the conformation of the disulphide bridges might influence the dissociation dynamics, as observed previously[5], but did not detect any correlation apart from the local environment imposing steric constraints.

The other amino acid containing a sulphur atom is methionine. Interestingly, we also observe a significant lengthening of the Cγ-Sδ bond in methionines but not of the Cε-Sδ bond (Figure 1d). It appears that the Cγ-Sδ bond dissociates and the S-methyl moiety moves away.

2.3 Protein backbone

In both proteins, positive and much weaker negative electron density is also apparent in the isomorphous difference maps $F_{obs(\Delta t)} - F_{obs(single\ pulse)}$[6] around the peptide bonds (see Figure 2). Positive difference electron density is situated close to the carbonyl oxygen atoms, away from the peptide bond, suggestive of a bond elongation. Interestingly, the presence of a strong positive peak seems to correlate with involvement of the carbonyl oxygen atom in a hydrogen bond. Peaks are missing e.g. in solvent exposed loops such as those around Asp60, Gly120 in thaumatin. This, however, could also be due to higher mobility. Negative difference electron density became clearly visible only after averaging the density of all peptide bonds (Figure 2b).

In contrast to the disulphide bonds, the atoms making up the protein backbone are light and the structural changes are small, complicating extraction of a statistically meaningful result. Therefore, we performed a jackknife analysis as described in the Methods section. The N-C_{alpha} and carbonyl C-O bond lengths increase significantly with time. It is very likely that their decrease at longer time delays, as the overall data quality deteriorates, is caused by automatic weighting favouring the geometry term over the X-ray term. Interestingly, this effect is barely noticeable for the N-C and C-C_{alpha} bonds (Figure 2d, Supplementary Fig. 13a). This cannot be explained by a systematic error, such as change in detector distance or wavelength, since the C_{beta}-C_{gamma} bond length also hardly changes with pulse length (Figure 2d).

We looked for a correlation between the observation of elongated bonds, secondary structure and B-factors. Loops and surface exposed regions of the protein backbone are expected to have higher B-factors and less well-defined electron density, potentially resulting in a bias towards the literature value of the bond geometry. Indeed, in general, the backbone carbonyl bond length is closer to the literature value for residues with high B-factors. This is clearly visible for the β-sheet and loop region of thaumatin (Supplementary Fig. 13b) and persists almost to the longest time delay. The effect is also visible for loop regions in lysozyme.Gd up to a time delay of 67 fs (Supplementary Fig. 13a). This clearly shows that the observed elongation of the averaged CO bonds is real and likely underestimated.

2.4 Aromatic side chains

All phenylalanine sidechains in thaumatine show strong negative electron density in the isomorphous difference maps $F_{obs(\Delta t)} - F_{obs(single\ pulse)}$, penetrating the ring perpendicular to the ring plane (Figure 3a). Much weaker negative difference density is observed in the phenyl rings of tryptophan residues (except Trp51) and in Tyr157 and Tyr169, both of which are solvent exposed. The other tyrosine residues do not show difference density. The different behaviour between the three aromatic residues is also apparent in the averaged electron densities (Figure 3b). The negative difference density is highest after a time delay of 18 fs and no longer visible at 54 fs. It is unclear whether this latter observation is an effect of data quality. These effects are similar in lysozyme.Gd but less obvious, possibly due to the more pronounced damage-induced loss in data quality.

2.5 Bound Gd

Due to their large interaction cross sections, heavy atoms predominantly undergo inner-shell photoionization, which changes their scattering properties. One might therefore expect to observe a decrease in their electron density. Since comparing absolute intensities is difficult, we compared the ratio of the integrated electron density of the Gd ions and a stretch of the protein backbone atoms as a function of pump probe time delay (Supplementary Fig. 14). This ratio does not change with time delay.

Supplementary Note 3

3. Computations analysing the complex mechanisms underlying X-ray induced dissociation of S-S bridges

3.1 Molecular dynamics simulations with XMDYN code

3.1.1 X-ray induced fast charging of constituent atoms

Simulations confirm the expected fast charging of the constituent atoms of the thaumatin sample, with the highest charge assigned to the heaviest atom, sulphur. Supplementary Fig. 15 shows the

predicted average ion charge for different X-ray fluences as a function of the time delay between pump and probe pulses. For each time delay, we extracted the values from the snapshots recorded at the time instant corresponding to the maximum intensity of the probe pulse. For the low fluence case F_{low}, the ionization dynamics of the non-hydrogen atoms are very similar. The average charge increases up to ~1, even higher for sulphur. With increasing X-ray fluence (Supplementary Fig. 15) the ionization increases and the charge evolution curves for light elements and sulphur deviate from each other more significantly, with the sulphur charge reaching values of +4 to +5. The increase in charge over time influences atomic dislocations in the irradiated thaumatin microcrystal. As observed experimentally (see Supplementary Note 2.2), the resulting displacements are the largest for the sulphur atoms, i.e., those atoms with the highest charge in thaumatin.

3.1.2 Effect of charged environment on the S-S separation

We performed molecular dynamics simulations at three different X-ray fluences (see Supplementary Discussion) testing different models in order to understand better the microscopic mechanisms influencing the S-S separation. First, the progressing separation of the sulphur ions in an isolated disulphide bridge were analysed in the absence of the atomic and electronic environment. Supplementary Fig. 16a-b shows the evolution of the S-S bridge in vacuum: (i) after irradiation with the X-ray pulse, and (ii) assigning the time-dependent charge from Supplementary Fig. 15 to the S atoms. In both cases, and for all fluence values considered, the unscreened Coulomb repulsion drives the S ions rapidly apart. Their relative displacement is much larger than deduced from the experimental data. Consequently, we conclude that the charged environment of the disulphide bridge influences its dynamics significantly, slowing the separation of the S ions.

Next the effect of positively and negatively charged S-S environments (created by ions and plasma electrons, respectively) were investigated separately on the S-S bridge dissociation. Supplementary Fig. 16d-f shows the evolution of the S-S pair in the thaumatin unit while (i) switching off the Coulomb interactions between the S atoms and free electrons, or (ii) switching off the Coulomb interactions between the S atoms and the non-S atoms. One can see that for all fluence cases the latter has a strong effect on the bridge dynamics. However, it is more pronounced for the medium and high fluence cases. In both cases, the predicted S-S separation is larger than

that derived from the experiment. At low fluence, it is comparable.

We interpret this in the following way: During and after exposure to the X-ray pulse, the increasingly charged S atoms begin to separate due to Coulomb repulsion. However, the repulsive force is reduced due to the presence of free electrons attracted by the S ions, which screen the interaction of the bare S ions. Nevertheless, the S-S separation would progress further were it not for the presence of charged non-S ions near the S-S bridge. When the S ions come too close to these non-S ions, the repulsive Coulomb force slows the S-S separation.

Supplementary Fig. 17 provides more detailed insight into the motion of the S-S pair. We plot there the total kinetic energy of the S-S pair, the translational energy (centre-of-mass energy) and the internal energy, corresponding to the vibrational or rotational motion. These are plotted for the maximum fluence case. The total kinetic energy of the disulphide bridge shows firstly a strong increase at time delays < 10 fs. This time interval corresponds to fast charging of the ions in the thaumatin unit cell (cf. Supplementary Fig. 15). Both S atoms quickly gain kinetic energy during this time. Afterwards, a rapid drop of the kinetic energy follows at delay times up to ~25 fs. This corresponds to the transient deceleration of the S atoms by the Coulomb repulsion to non-S atoms, giving rise to the plateau in displacement of Figure 4. Concurrently the internal energy of the S-S pair (which initially strongly increases) as well as the translational energy (which increases several fs later than the internal energy), decrease during this time. The kinetic energy is regained from the potential energy at ~50 fs delay and remains then almost constant, indicating a further increase of the S-S separation and finally leading to a randomization of the S ions' motion, in accordance with the trends seen in Figure 4.

During the entire evolution, the corresponding curves for the translational and internal energies show damped oscillatory behaviour, and are partially out of phase. This indicates that the energies partly interchange, reflecting various transient translational and vibrational/rotational modes within the complex trajectory of the S-S pair. This confirms the strong effect of the charged environment on the dynamics of the ionized disulphide bridge, in marked contrast to its behaviour in vacuum (cf. Supplementary Fig. 16), when the Coulomb forces only induce a progressive linear separation of the S ions.

3.2 Hybrid model of molecular dynamics and plasma physics

Here we report further information about the lysozyme crystals performed with the hybrid model simulations. The key results are shown in Fig. 4 of the main article. In the rate equations calculation all of the trapped electrons are assumed to thermalize on a sub-femtosecond time scale. The energy distribution is accordingly Maxwell–Boltzmann but the mean temperature changes with time (Local Thermodynamic Equilibrium condition). The thermalization assumption appears to generate higher charge states than the XMYDN calculation (see Supplementary Fig. 15). For a consistent comparison between the two theory models, results for the hybrid model used charge states that closely matched the XMDYN simulations after adjustment for the difference in the number of pulses simulated (XMDYN simulations modelled both pump and probe pulses). The final adjusted charge states used in the hybrid model are shown in Supplementary Fig. 18.

Typically, the asymptotic value of the Debye length is calculated to lie in the range 1-10 Å under the presumed experimental illumination conditions; the value $\lambda_D = 2$ Å has been used in the ion dynamics simulations reported here. If the asymptotic Debye length exceeds 10 Å, electrostatic repulsions induce sufficiently large amplitude motion of the ions to rapidly disrupt the structure. For the value of Debye length that is obtained from the plasma simulations, the contribution of ions located further than 20 Å from a given atom to the net force acting on it is less than 1% due to an exponential decay of the screened electrostatic interaction.

Supplementary Discussion

Comparison of experimental and theoretical results - Effect of the unknown effective fluence distribution

A reliable estimate of the operative X-ray fluence absorbed by the sample needs to be available for a meaningful analysis of experimental data and subsequent comparison with specific theoretical predictions. For the crystalline samples considered here, with crystal comparable in size to the beam focus and small in comparison to the jet diameter, the effective fluence may strongly differ from its nominal (experimentally determined) value. This results from an intrinsic uncertainty in location of sample relative to beam focus during exposure. One can only determine

spatially averaged values of measured observables. Even in that case, however, the spatial distribution of fluence should be known as accurately as possible. Whereas the spatial distribution of the X-ray beam can to good approximation be described as a Gaussian (or double Gaussian) distribution, the distribution of crystals within the jet is stochastic and so *per se* unknown. In addition, the crystals invariably have a distribution of sizes. Their size being comparable to that of the beam focus, the crystals might be irradiated directly by the centre of the X-ray beam, marginally grazed by one of its wings, or anything in between. A significantly different absorbed dose is delivered accordingly, inducing a correspondingly different degree of sample ionization and constituting yet another uncertainty in irradiation conditions for a given XFEL pulse.

Insofar as estimation of the nominal fluence is concerned, the experimental determination is not straightforward and the accuracy is influenced by many factors. The focal size and characteristics can change with modifications of the machine operation/beam parameters and with instrumental drift during the experiment. Beamline transmission and focussing properties of the mirrors are often not well characterized. The spatial and temporal distribution of the photons in an XFEL pulse, as well as the pulse energy, can change from shot to shot. While the latter is measured and known on every pulse, there is no shot to shot measurement of the spatial profile. Some limited information on the temporal profile of the beam can be extracted from the XTCAV measurements. For these reasons there is a large degree of uncertainty in the values of these parameters, . all of which influence the irradiation of the sample.

The fluence values we report in the paper are nominal values. In view of the discussion above, the effective fluence values are likely lower than reported in Supplementary Table 1. We therefore performed the theoretical analysis of ionization dynamics at three different fluence values, with the highest one corresponding to the nominal fluence value measured in the experiment.

Other factors can also affect the observed response of the crystals in comparison with the theoretical analysis, notably the information convolution inherent to recorded diffraction images. Given Bragg termination of the diffraction signal, the measured high resolution diffraction will contain a large contribution from still relatively undamaged protein. This, in fact, makes SFX possible. For experiments geared at analysing light-matter interaction, however, it becomes a

complicating factor (and may be a major concern for single particle imaging, for which diffraction is aperiodic and hence cannot be 'gated' in the sense of Bragg diffraction). All of these factors can contribute to a quantitative discrepancy between the experimental and theoretical results. Nonetheless the two different theoretical models agree as to the observed displacements of the sulphur ions with time, inspiring a certain confidence in the results.

Supplementary References

1 Barty, A. *et al.* Self-terminating diffraction gates femtosecond X-ray nanocrystallography measurements. *Nat. Photonics* **6**, 35-40 (2012).

2 Lomb, L. *et al.* Radiation damage in protein serial femtosecond crystallography using an x-ray free-electron laser. *Phys. Rev. B* **84**, 214111 (2011).

3 Nass, K. *et al.* Indications of radiation damage in ferredoxin microcrystals using high-intensity X-FEL beams. *J. Syn. Rad.* **22**, 225-238 (2015).

4 Wong, J. W. H. & Hogg, P. J. Analysis of disulfide bonds in protein structures. *J. Thromb. Haemost.* **8**, 2345 (2010).

5 Gerstel, M., Deane, C. M. & Garman, E. F. Identifying and quantifying radiation damage at the atomic level. *J. Syn. Rad.* **22**, 201-212,

6 Bury, C. S. & Garman, E. F. RIDL: a tool to investigate radiation-induced density loss. *J. Appl. Cryst.* **51**, 952-962 (2018).

7 Weierstall, U., Spence, J. C. H. & Doak, R. B. Injector for scattering measurements on fully solvated biospecies. *Revi. Sci. Instr.* **83**, 035108 (2012).

8 Ferguson, K. R. *et al.* Transient lattice contraction in the solid-to-plasma transition. *Sci. Adv.* **2**, e1500837 (2016).

9 Engh, R. A. & Huber, R. Accurate bond and angle parameters for X-ray protein structure refinement. *Acta. Cryst.* **A47**, 392-400 (1991).

Supplementary Figures

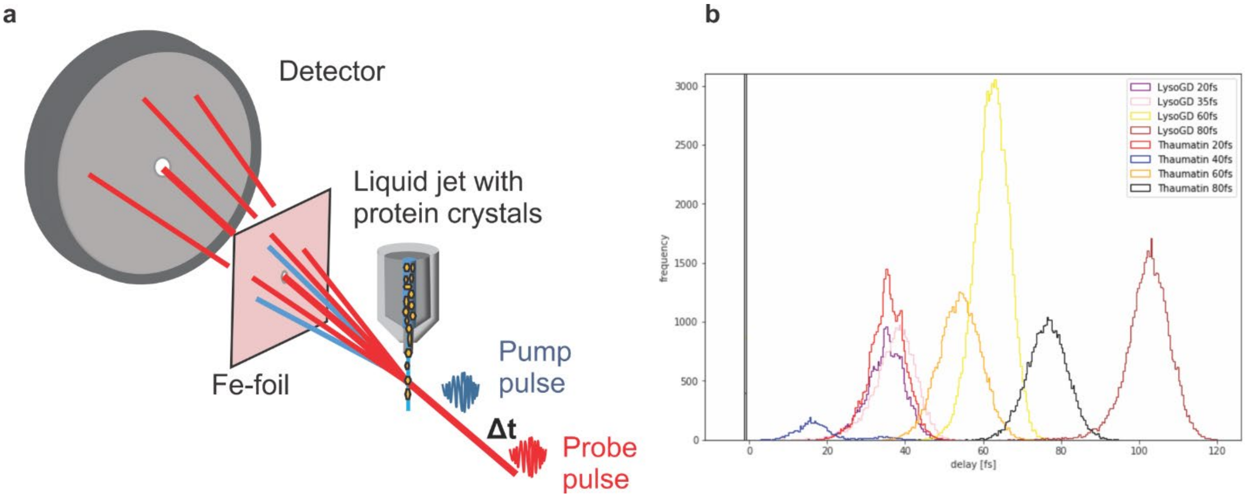

Supplementary Figure 1: Experimental setup
Protein nanocrystals were injected into the XFEL beam using a gas dynamic virtual nozzle injector[7]. The first ~15 fs X-ray pulse, with photon energy above the iron K-edge (7.112 keV) and a pulse energy of ~0.5 mJ was used as a pump, inducing ionization dynamics in the system. The scattered X-rays were absorbed by an iron filter (thickness 25 µm) and did not reach the detector. After a certain time delay (20 fs $\leq \Delta t \leq$ 100 fs) a second 15 fs X-ray pulse, with a photon energy just below the iron K-edge and a pulse energy of ~0.5 mJ, was used as a probe to measure Bragg diffraction, hitting the same sample segment. In this case, the scattered X-rays passed through the iron filter. The setup is similar to the one used to probe the X-ray induced solid-to-plasma transition in Xenon nanoclusters[8]. **b**) Histogram of XTCAV values.

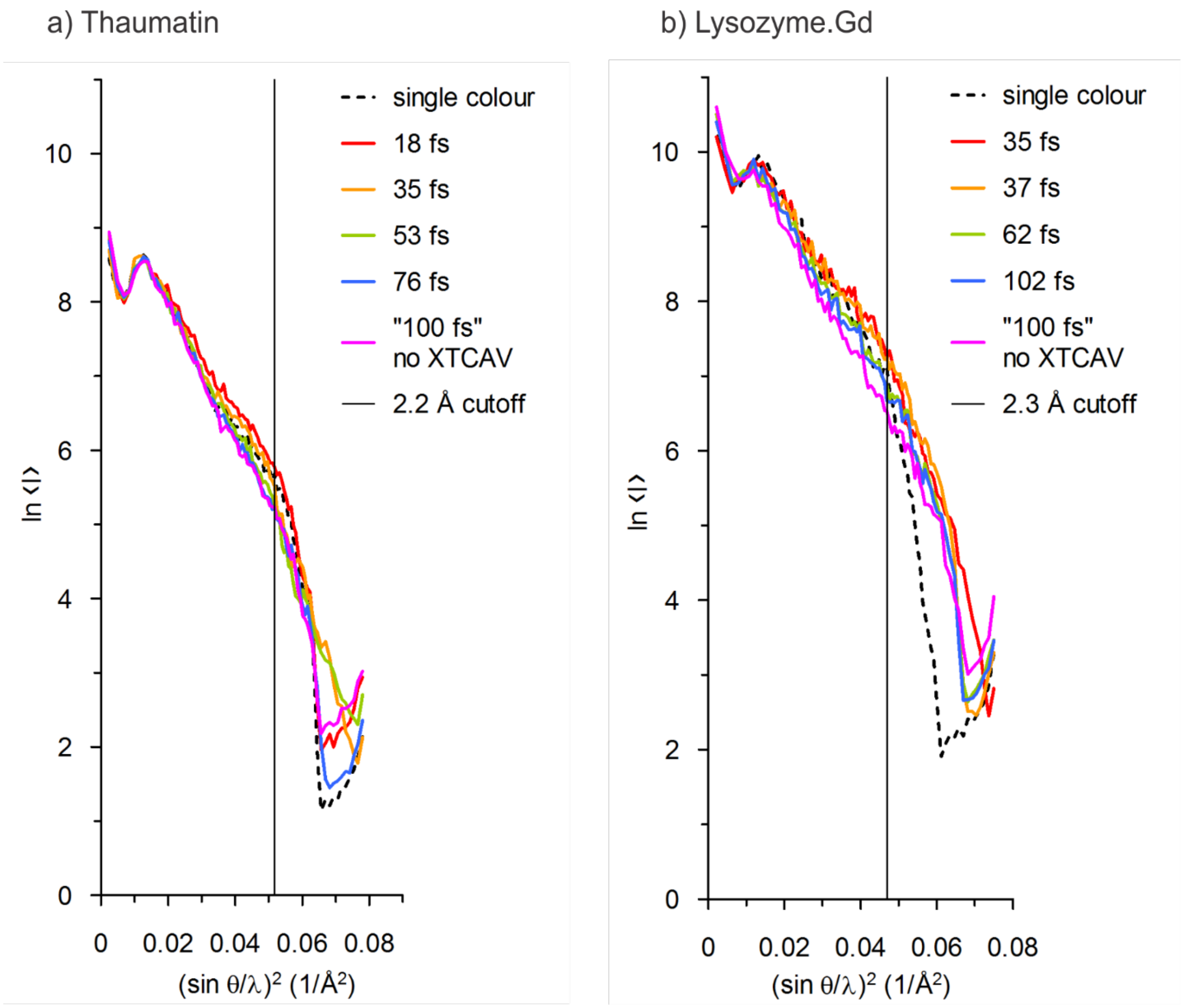

Supplementary Figure 2: Wilson plots of thaumatin (a) and lysozyme.Gd (b) SFX data. Wilson plots were calculated directly from the CrystFEL intensities as $ln\langle I_{obs}\rangle vs \left(\frac{sin\theta}{\lambda}\right)^2$ in 80 resolution bins, and shifted along the y-axis to minimize the least-squares error between the curves for the first 10 data points only. The effects of shadowing the detector by the injector shroud is clearly visible for the single pulse Lysozyme.Gd data. An increase of the Debye-Waller factor (B-factor) with pump probe delay is clearly apparent (**a, b**) and more pronounced for the Lysozyme.Gd (**b**).

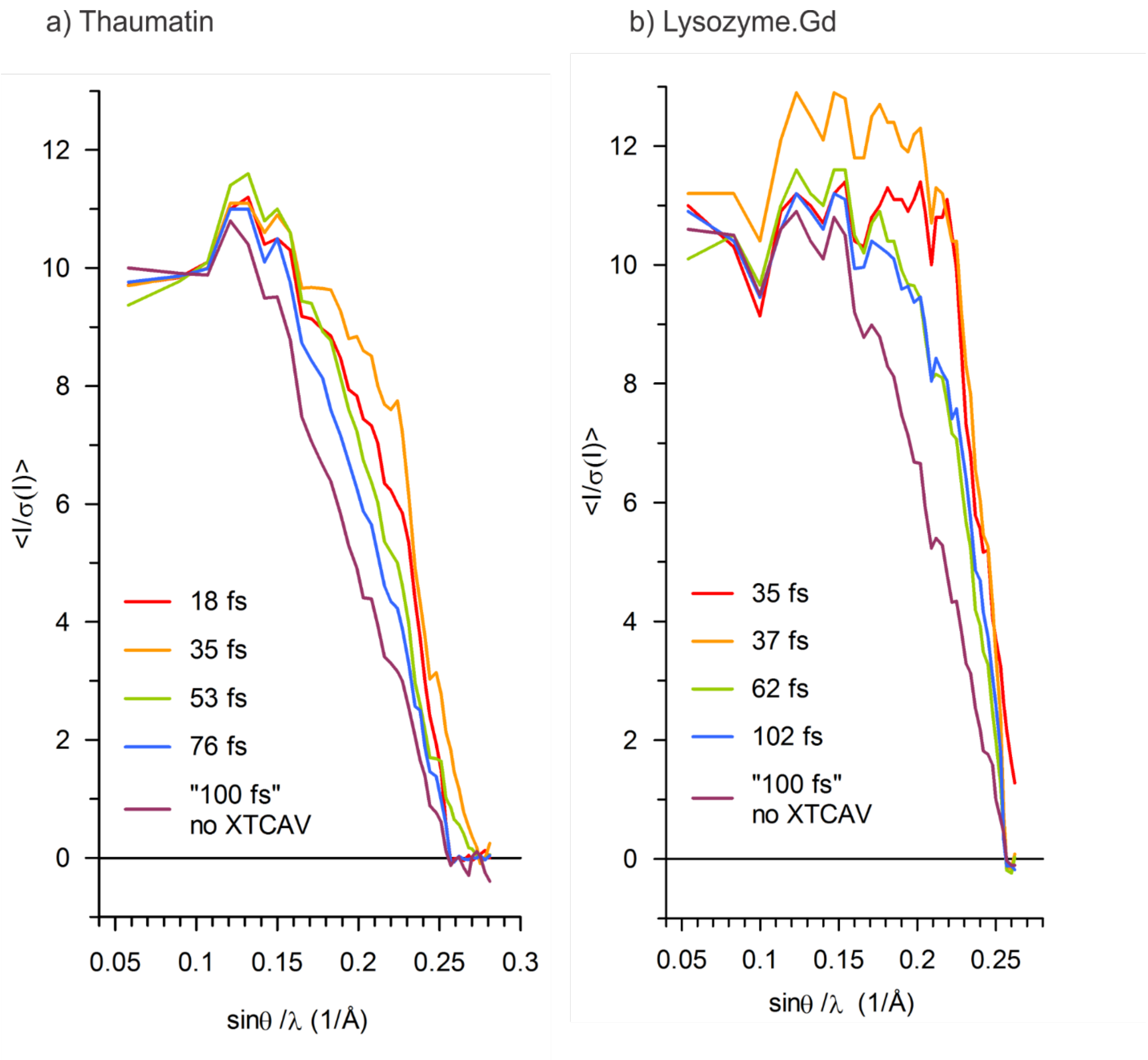

Supplementary Figure 3: **I/σ(I) as a function of pump probe time delay**. Values were calculated using the Monte-Carlo integrated intensities and their σ values as calculated by CrystFEL. **a**) thaumatin SFX data, **b**) lysozyme.Gd SFX data.

(**a**) N(Z) statistics**:** Thaumatin

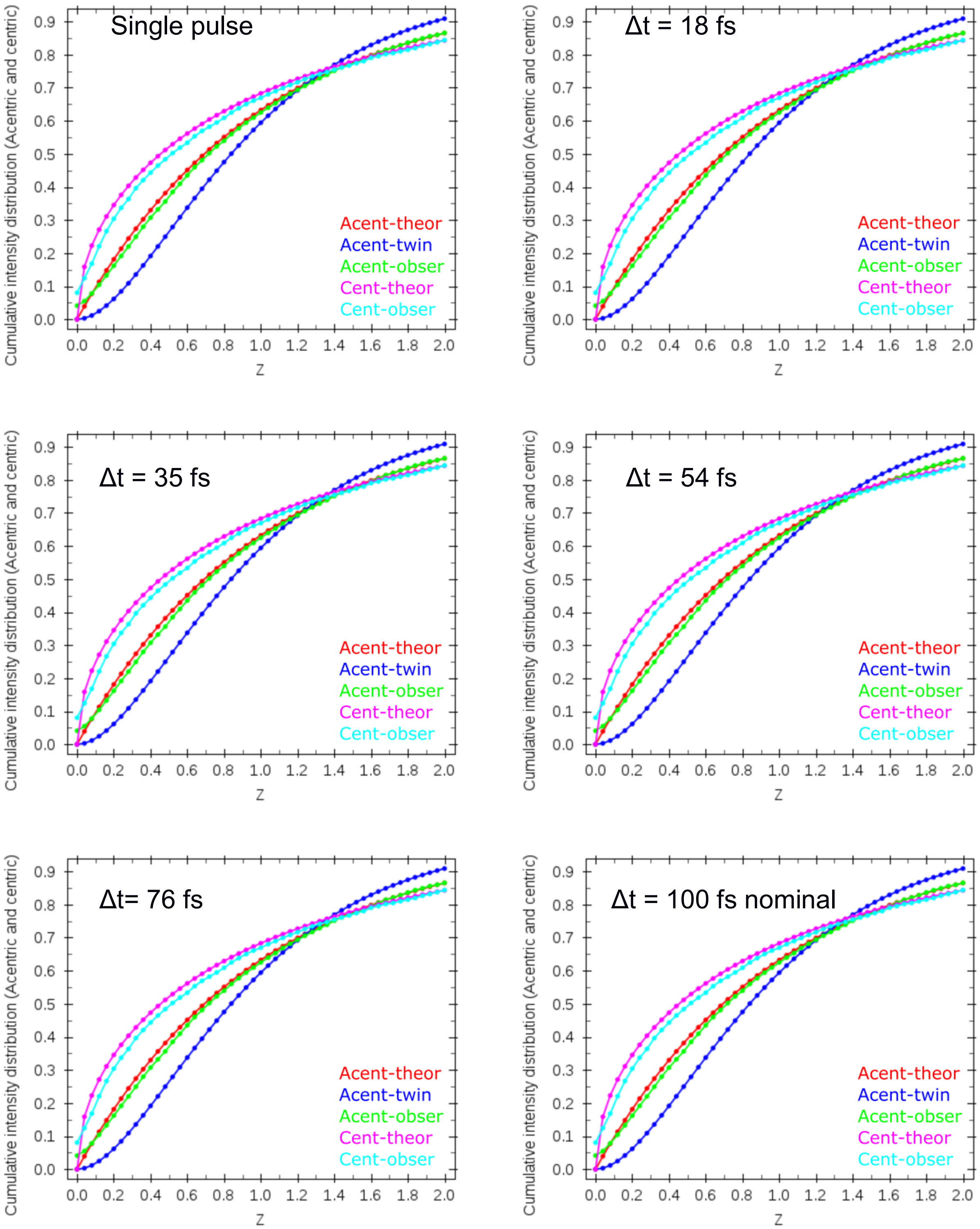

(**b**) N(Z) statistics**:** lysozyme.Gd

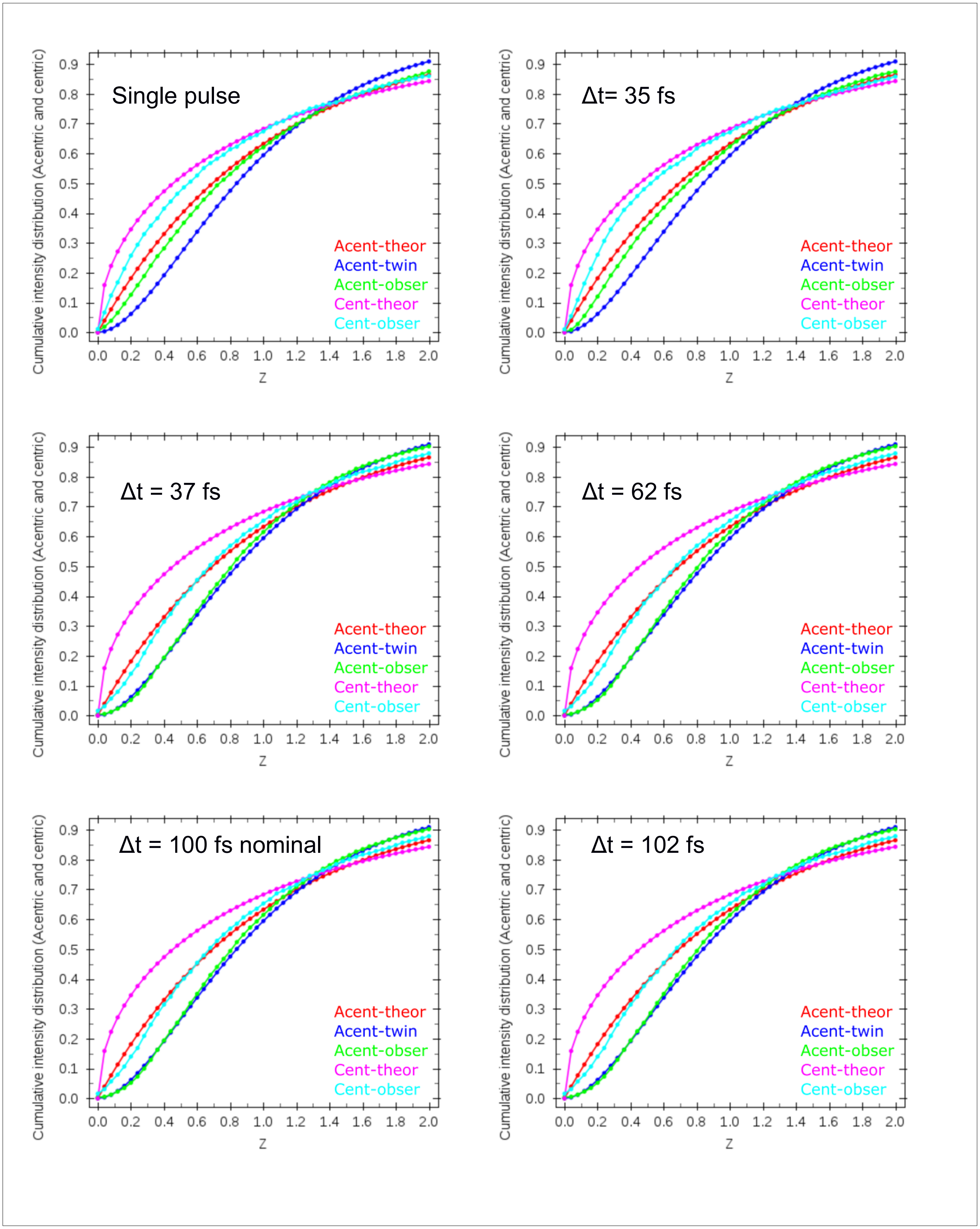

Supplementary Figure 4: Cummulative Intensity distributions (N(Z). The distribution of the thaumatin data (**a**) is as expected, whereas the lysozyme.Gd data (**b**) show increasing deviations from the expected distribution with longer pump probe time delays. The reason is likely the higher dose due to the presence of 100 mM Gd.

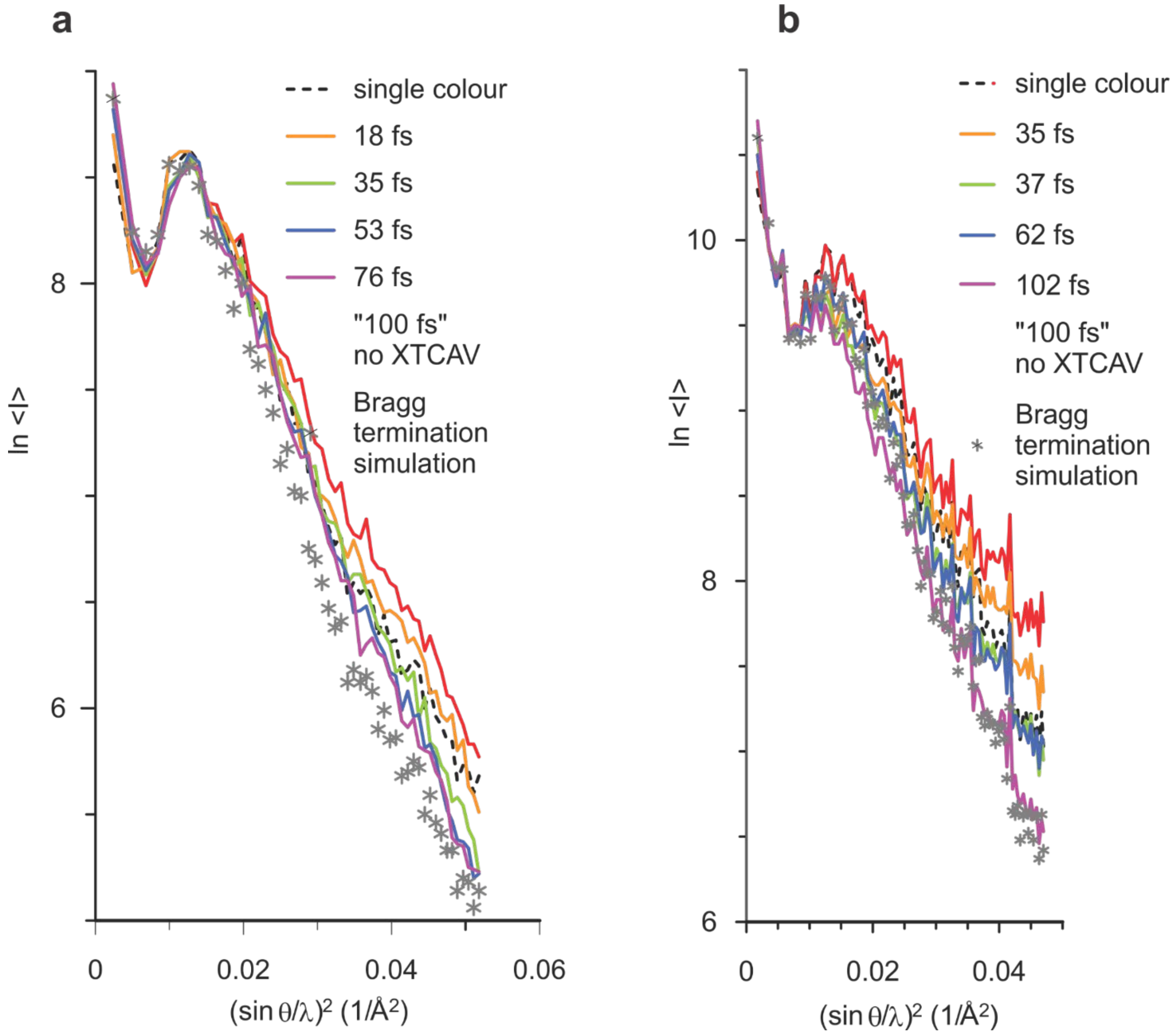

Supplementary Figure 5: Simulation of Bragg Termination.

We simulated the effect of Bragg termination[1] by modifying the Bragg intensities of the single pulse data according to Barty *et al.*[1]. Since we cannot simulate the effect of the "evolution" time between pump and probe pulse on the sample, we assumed a probe pulse length of 100 fs. Specifically, the single-pulse intensities of thaumatin (**a**) and lysozyme.Gd (**b**) were modified by applying increasing B-factors in a step-wise fashion, and summing the resulting intensities:

$$I_{hkl}^{Bragg\ termination} = \sum_0^n I_{hkl}^{single\ color} e^{-2B_{eff}\frac{\sin^2\theta}{\lambda^2}} \quad (14)$$

where $B_{eff} = \left(\frac{n}{20.0}\right)^3$ (15)

This simulates the increase in B factor with the third power of time during the XFEL pules[1]. For thaumatin, 100 steps were required to obtain results comparable to the observed data for 100 fs time delay, whereas 160 steps were needed for lysozyme to reproduce the Wilson plots observed for the longest pump probe time delay.

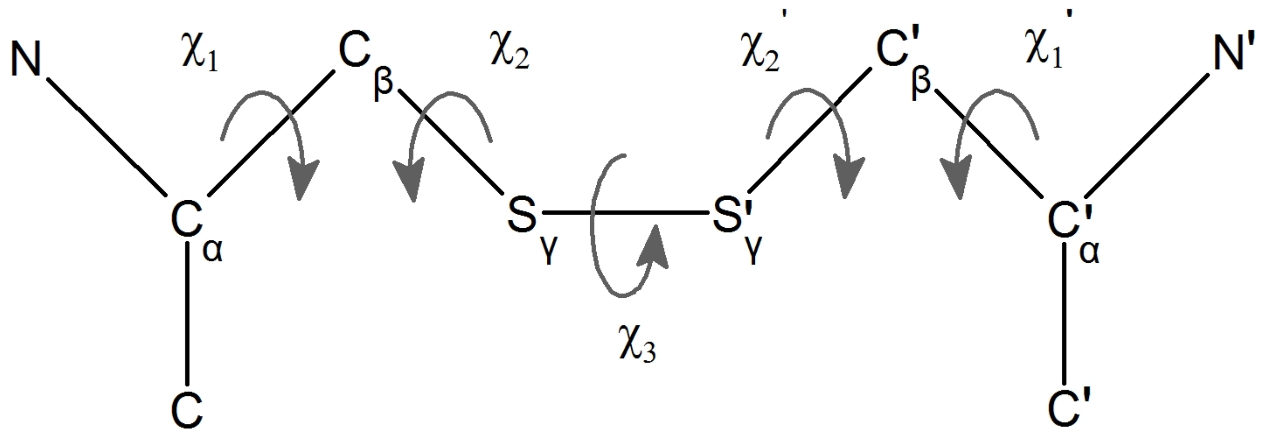

Supplementary Figure 6: Geometry and nomenclature of a disulphide bond

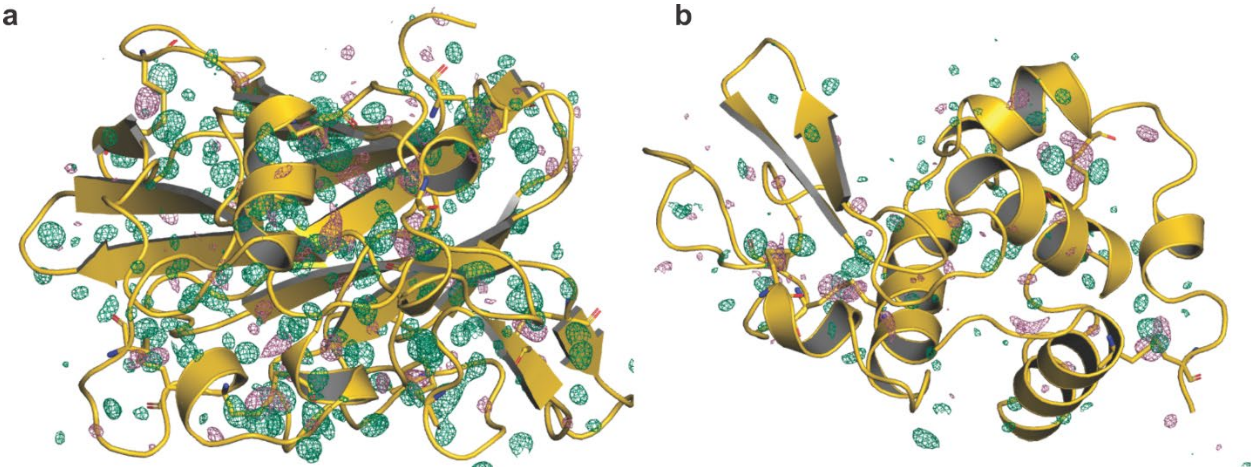

Supplementary Figure 7: Differences densities in thaumatin (a) and lysozyme.Gd (b). Isomorphous difference electron density maps ($F_{obs(\Delta t)}$ $-F_{obs(single\ pulse)}$) of the two proteins contoured at -3σ (pink) and +3σ (green) show difference density peaks around all disulphide bonds. This effect is independent of secondary structure, thaumatin contains a large β-sheet, lysozyme is mainly α-helical. The negative (pink) peak in the middle of the S-S bond and the two positive peaks (green) along the bond direction are indicative of an elongation of the disulphide bonds. Most of the additional positive difference peaks in thaumatin are close to backbone carbonyl oxygen atoms (see Figure 2, Supplementary Fig. 12). The effect is strongest for Δt= 18 fs for thaumatin (**a**), The difference in time delay (Δt= 18 fs for thaumatin (**a**) and 35 fs for lysozyme.Gd (**b**) may be the reason that there are fewer peaks in lysozyme.Gd (**b**).

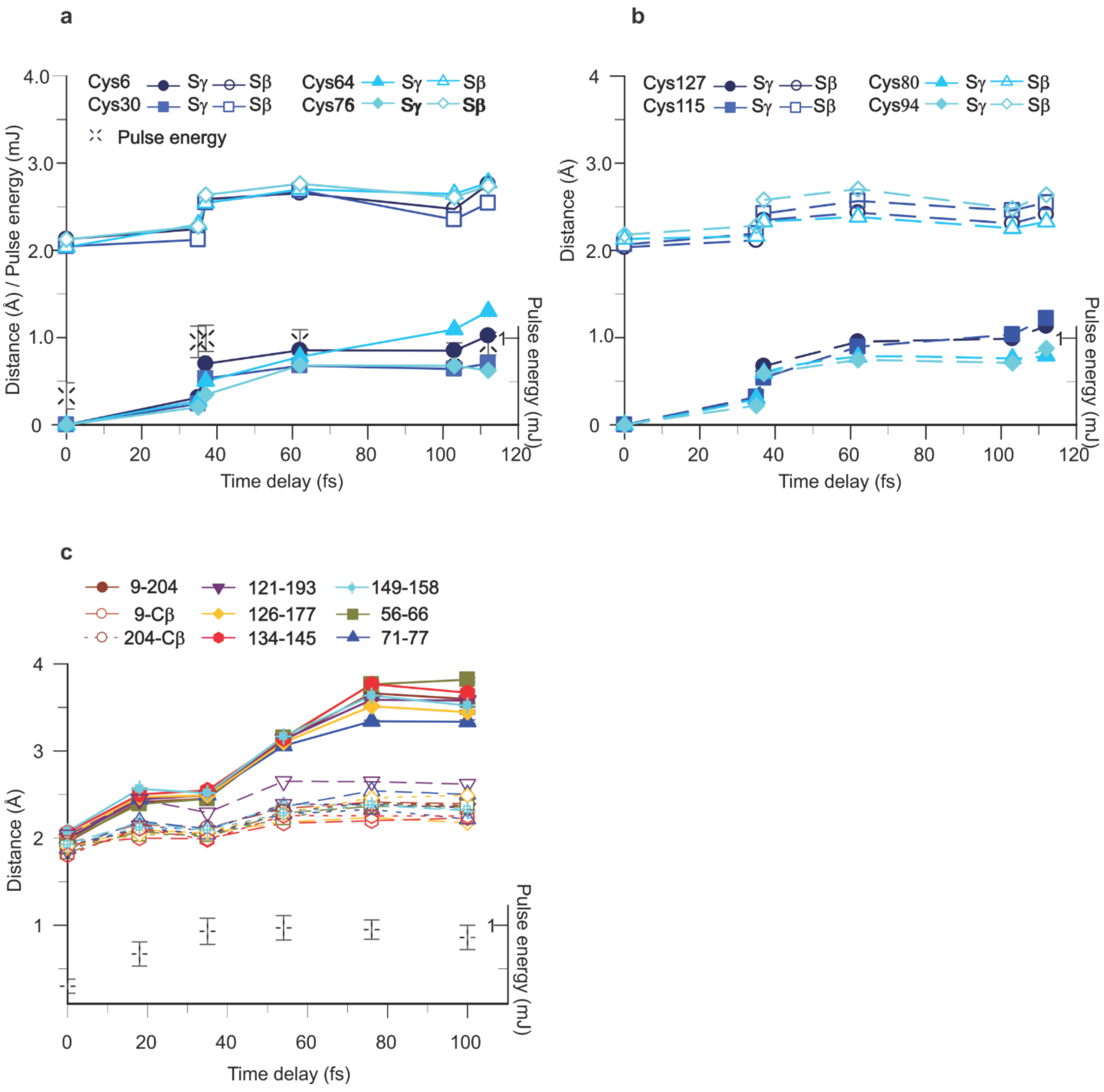

Supplementary Figure 8: Changes in S-S, Sγ-Cβ distances. **a**,**b** Lysozyme.Gd. The relative displacements of the sulphur ions from their location in the single pulse data is plotted along with the increase of the **S**γ-Cβ distance. The two bonds belonging to a disulphide bond are displayed separately. **c**) Thaumatin. The elongation of the S-S distance is plotted as a function of pump probe delay time (see Fig. 1c for lysozyme.Gd) along with the increase of the **S**γ-Cβ distance. The two values for each S-S bond are averaged. The sequence numbers of the cysteine residues are given in the figure legend. The joint pulse energy of the pump and probe pulses is also shown. The error bars correspond to the standard deviation.

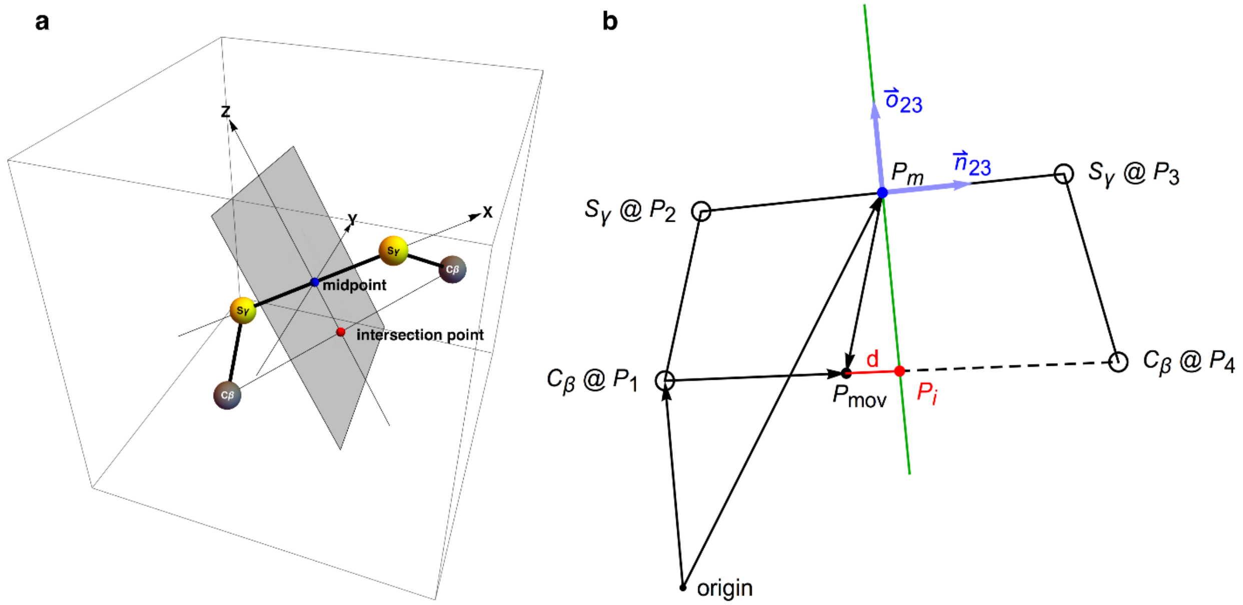

Supplementary Figure 9: Local coordinate system for the disulphide bridges. (a) Sketch of local coordinates chosen to characterize the disulphide bridge. A right-handed orthogonal coordinate system is delineated by first defining two characteristic points, the “midpoint” P_m and the “intersection point” P_i. The midpoint is situated halfway along the line connecting the sulphur atoms. A plane is passed, normal to this line, through the midpoint. The intersection point of this plane with the line connecting the respective Cβ atoms defines the intersection point. (**b**) Definition and nomenclature of characteristic points and vectors for local coordinates in the disulphide bridge. The unit vectors $\mathbf{n}_{23}$ and $\boldsymbol{\sigma}_{23}$ that define the x and y directions are described in Supplementary Note 1.

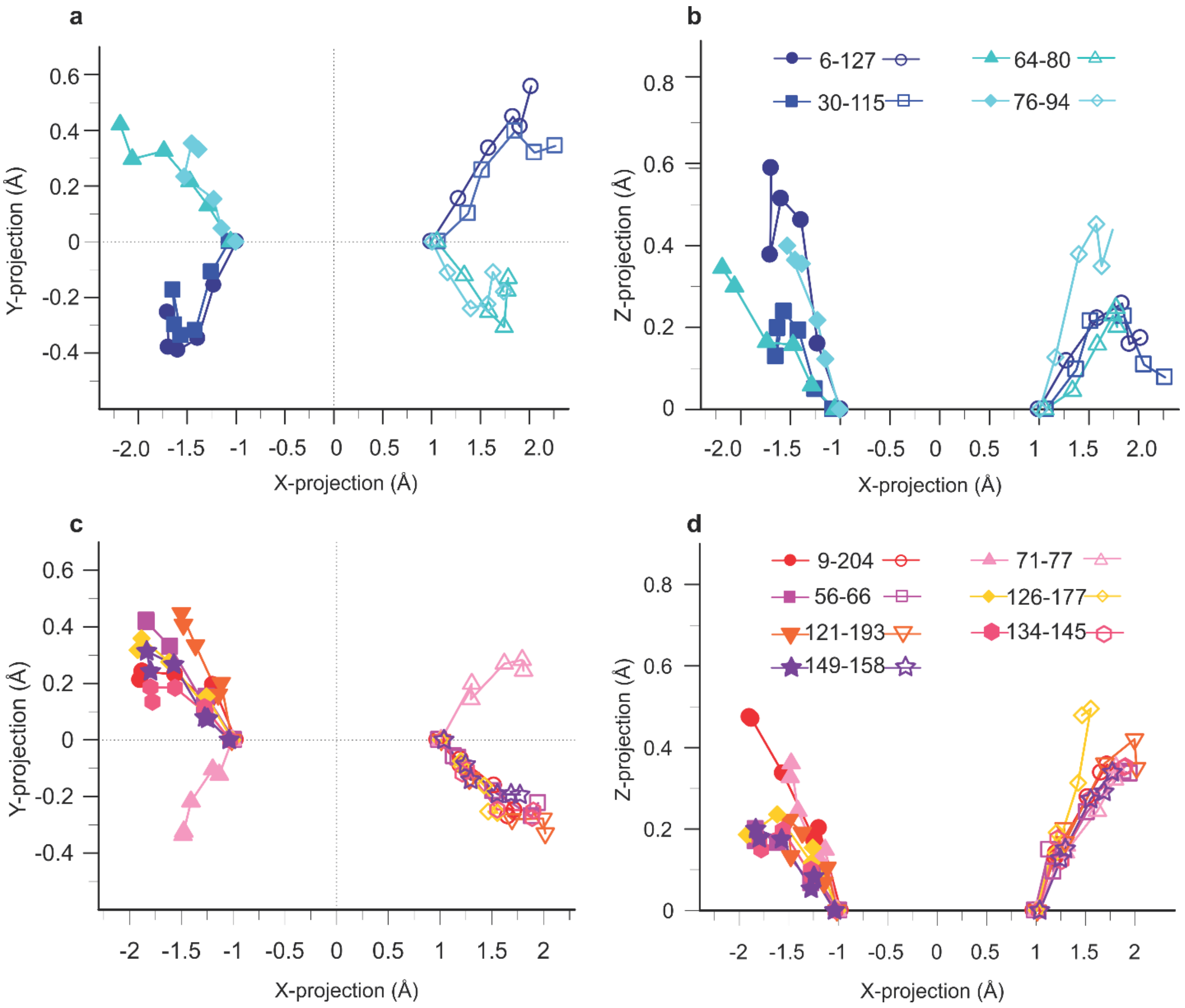

Supplementary Figure 10: Sulphur ion trajectories in lysozyme.Gd (a,b) and thaumatin (c,d). 2D projections of the (linearly interpolated) sulphur ion trajectories in a local coordinate system of the disulphide bridge (Supplementary Fig. 9). Trajectories of sulphurs ions originating from a disulphide bond are plotted in matching colours and symbols. The sequence numbers of the cysteine residues are given in the figure legend.

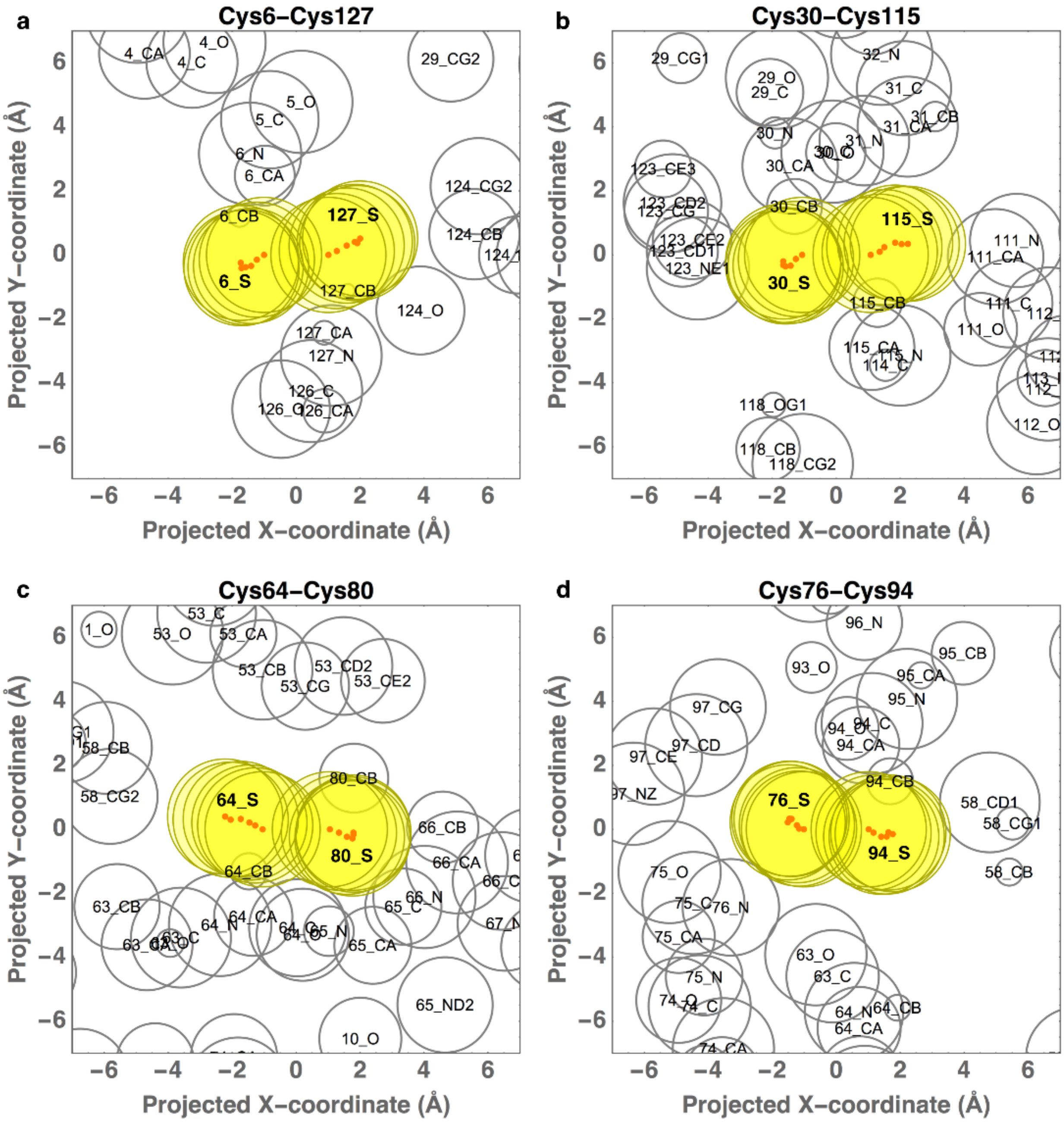
a
Cys6–Cys127
b
Cys30–Cys115
c
Cys64–Cys80
d
Cys76–Cys94
Projected X–coordinate (Å)
Projected Y–coordinate (Å)
4_CA
4_O
4_C
29_CG2
5_O
5_C
6_N
6_CA
124_CG2
6_CB
127_S
124_CB
6_S
127_CB
124_O
127_CA
127_N
126_C
126_CA
29_CG1
32_N
29_O
29_C
31_C
31_CB
31_CA
30_N
31_N
30_CA
123_CE3
30_CB
115_S
123_CE2
123_CD1
123_NE1
111_N
111_CA
30_S
115_CB
111_C
111_O
115_CA
114_C
118_OG1
112_O
118_CB
118_CG2
1_O
53_C
53_O
53_CA
53_CB
53_CD2
53_CG
53_CE2
58_CB
58_CG2
64_S
80_CB
66_CB
80_S
66_CA
64_CB
66_N
63_CB
64_CA
65_C
65_N
65_CA
65_ND2
10_O
96_N
95_CB
93_O
95_CA
95_N
97_CG
94_C
94_CA
97_CD
97_CE
94_CB
58_CD1
76_S
58_CG1
94_S
75_O
58_CB
75_C
76_N
75_CA
63_O
75_N
63_C
64_CB
64_N
64_CA
74_CA

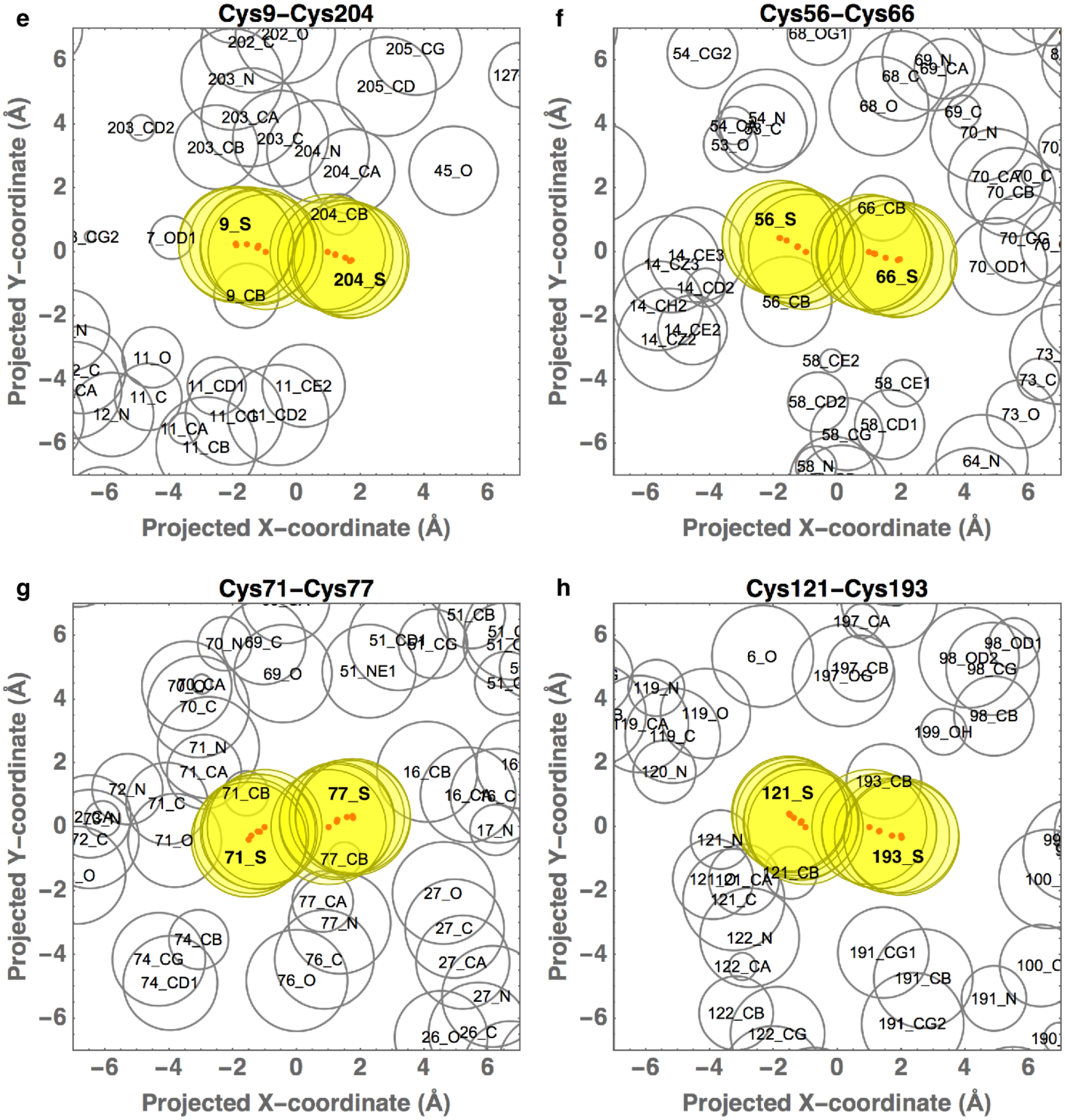

e
Cys9–Cys204
9_S
204_S
f
Cys56–Cys66
56_S
66_S
g
Cys71–Cys77
71_S
77_S
h
Cys121–Cys193
121_S
193_S
Projected X-coordinate (Å)
Projected Y-coordinate (Å)

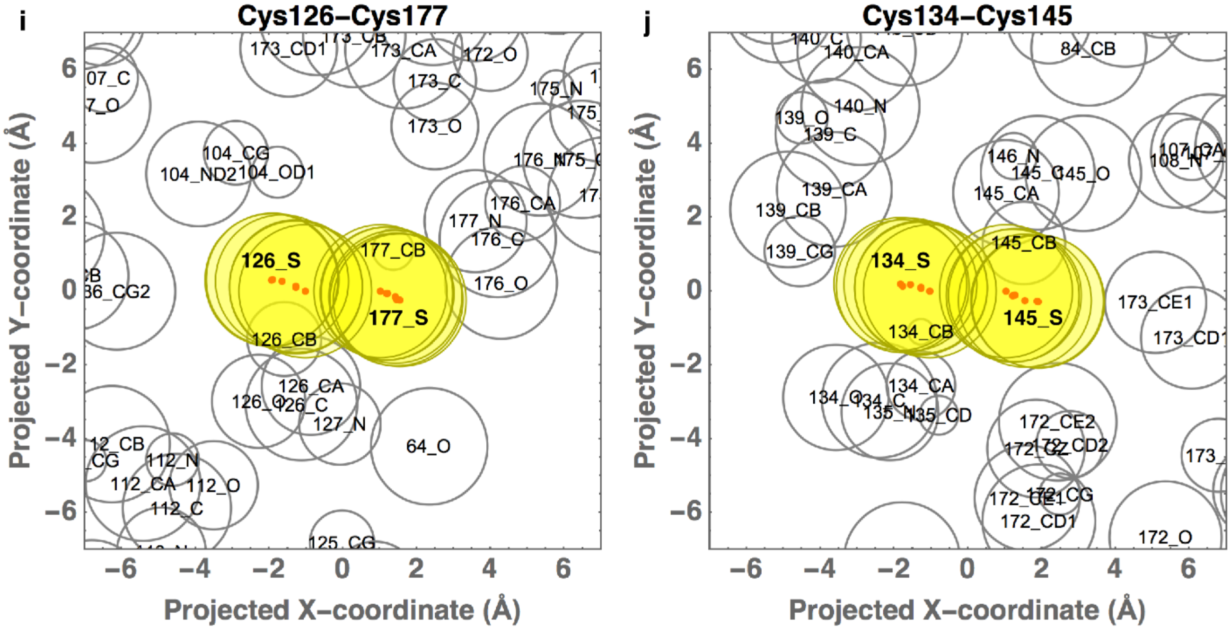

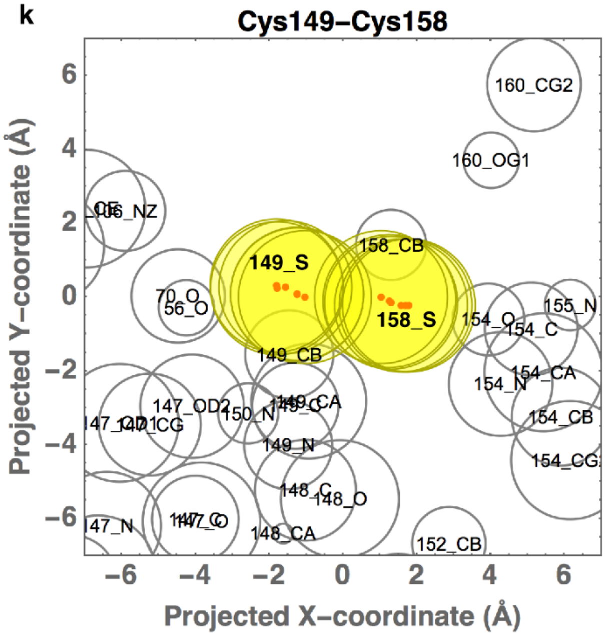

Supplementary Figure 11. Steric environment of the cysteine sulphur ions. (**a-d**) shows the view in the X-Y plane of local coordinates (see Supplementary Fig. 9,10) in lysozyme.Gd. The trajectories of the sulphurs of Cys6 (**a**) and Cys94 stop, they are facing the solvent; Cys80 (**c**) and Cys30 (**b**) stop because of steric hindrance. Cys127 (**a**) moves into a protein void. (**e-k**) shows the **s**teric environment of the cysteine sulphur ions (in the X-Y plane of local coordinates (see Supplementary Fig. 9,10) in thaumatin. A radius of 1.8 Å was used for all atoms.

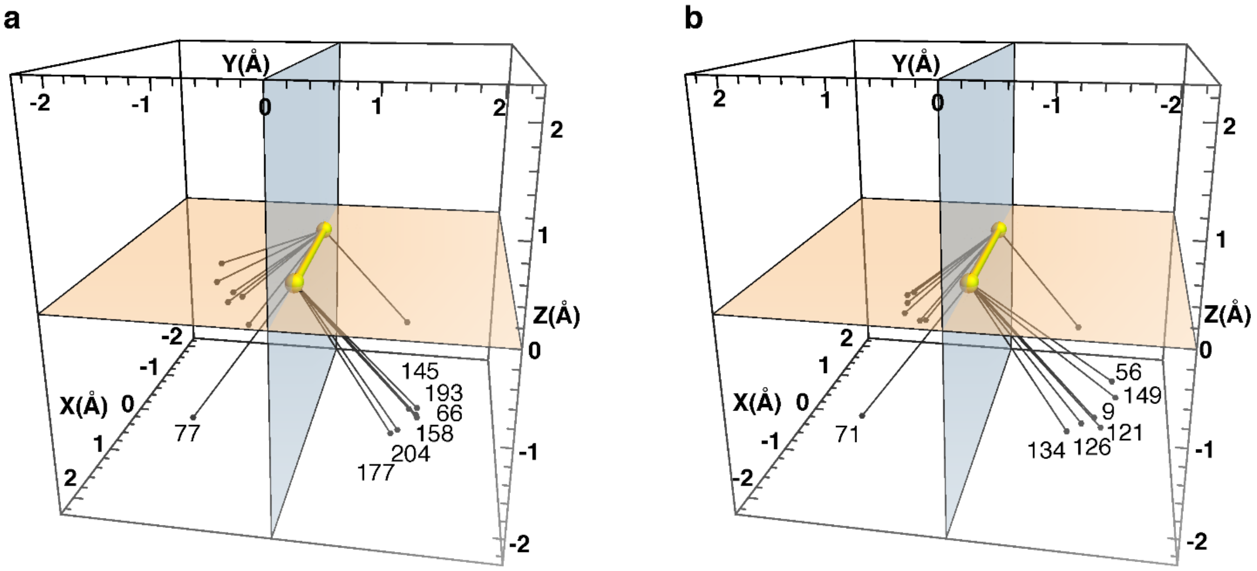

Supplementary Figure 12: 3D plot of the Cβ-Sγ-Sγ-Cβ atom positions in thaumatin at different pump probe delays. The X-Y plane is shown in beige (see Supplementary Fig. 9). The Cβ atoms in the lower right quadrant in **a** (front view) have a narrower spatial distribution than the other (see **b** back view). This is also apparent in the 2D projection shown in Supplementary Fig. 10.

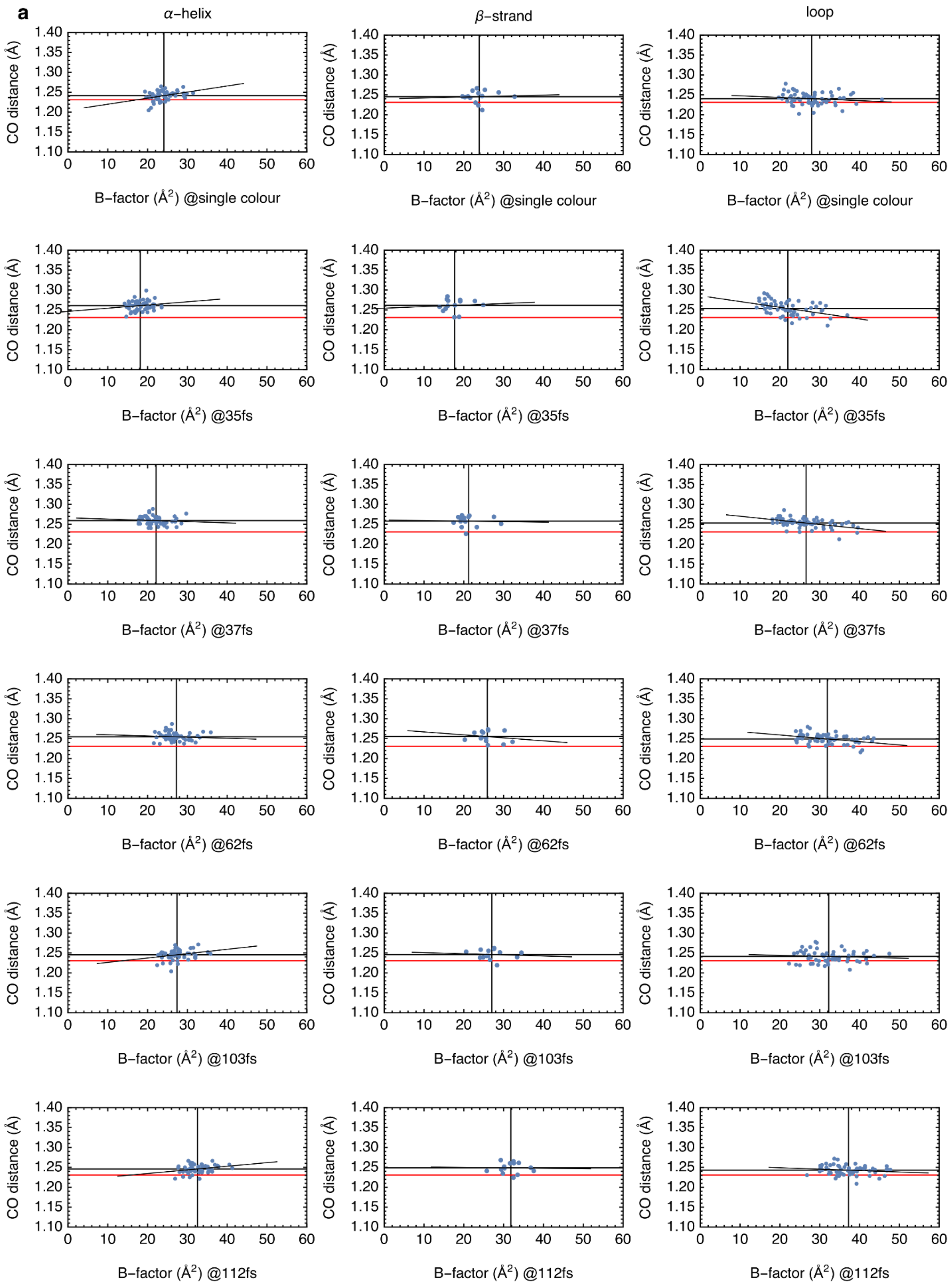
a
α−helix
β−strand
loop
CO distance (Å)
B−factor (Å^2) @single colour
B−factor (Å^2) @35fs
B−factor (Å^2) @37fs
B−factor (Å^2) @62fs
B−factor (Å^2) @103fs
B−factor (Å^2) @112fs

b

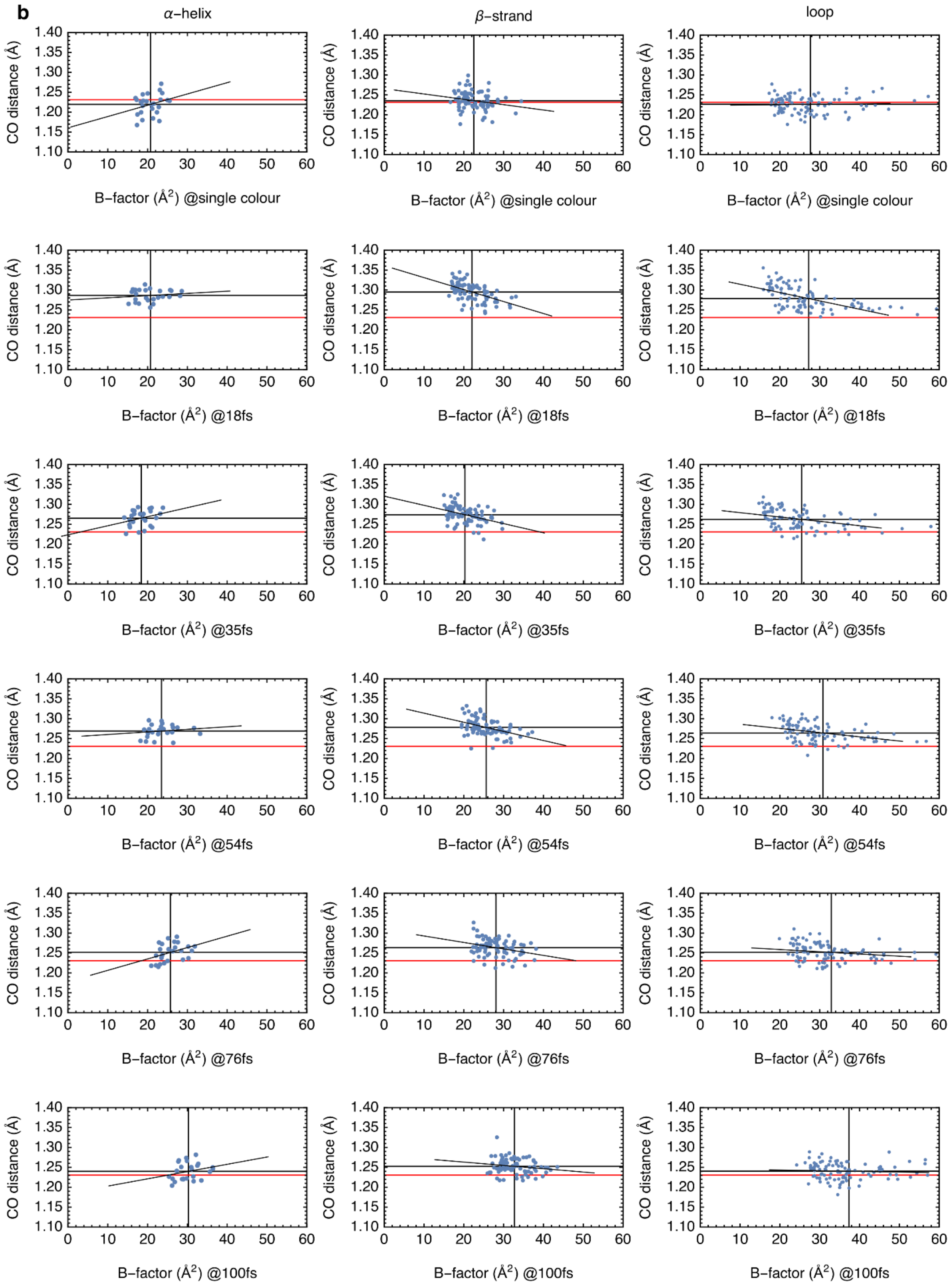

α−helix
β−strand
loop
CO distance (Å)
B−factor (Å^2) @single colour
B−factor (Å^2) @18fs
B−factor (Å^2) @35fs
B−factor (Å^2) @54fs
B−factor (Å^2) @76fs
B−factor (Å^2) @100fs

Supplementary Figure 13: Plot of C=O bond length as a function of secondary structure of the protein (a lysozyme.Gd, b thauamtin) and pump probe time delay. Lysozyme (**a**) contains mainly α-helices, thaumatin (**b**) predominantly β-strands. In both proteins the backbone carbonyl (C=O) bond length refines to the reference value[9] (indicated by the red line) in case of the single pulse data. In contrast, for the pump probe data the C=O bond length refines to a larger value. This is not due to a decrease of data quality and increase of disorder (high B-factors). In contrast, for the longer time delays (increase of disorder, decrease of data quality, see Supplementary Fig. 2-5, Supplementary Table 1,2), the geometry term dominates refinement and the C=O bond length approaches the reference value again. This effect is much stronger for lysozyme, most likely due to higher ionization caused by the presence of 100 mM Gd. The black lines indicate the refined mean values of the B-factor and backbone C=O bond length. The single pulse mean B-factors are relatively high due to lower data quality (see Supplementary Table 1,2) due to lower pulse energy and a shadowing of the detector by the injector shroud.

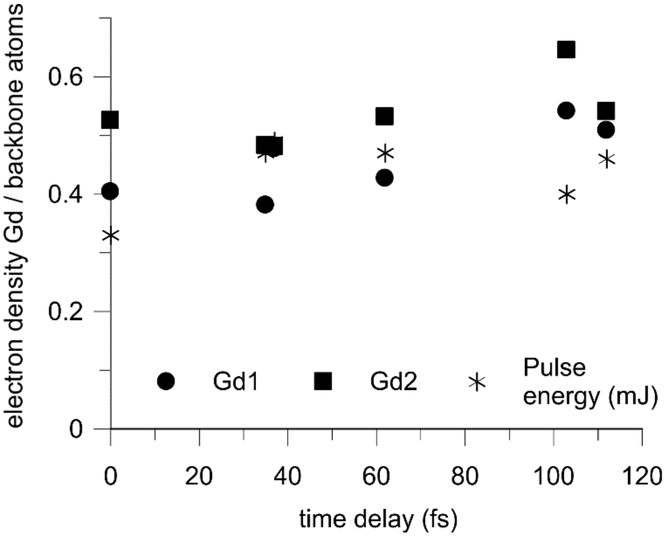

Supplementary Figure 14: Electron density of the Gd ions as a function of pump probe delay time. The electron density of the Gd ions was normalized by calculating the ratio of the integrated electron density of the Gd ions (1.0 Å integration radius) and a stretch of the protein backbone atoms (residues 50-59, 1.5 Å radius). The ratio of the electron densities seams rather stable. The measured pulse energy of pump and probe pulses was divided by two.

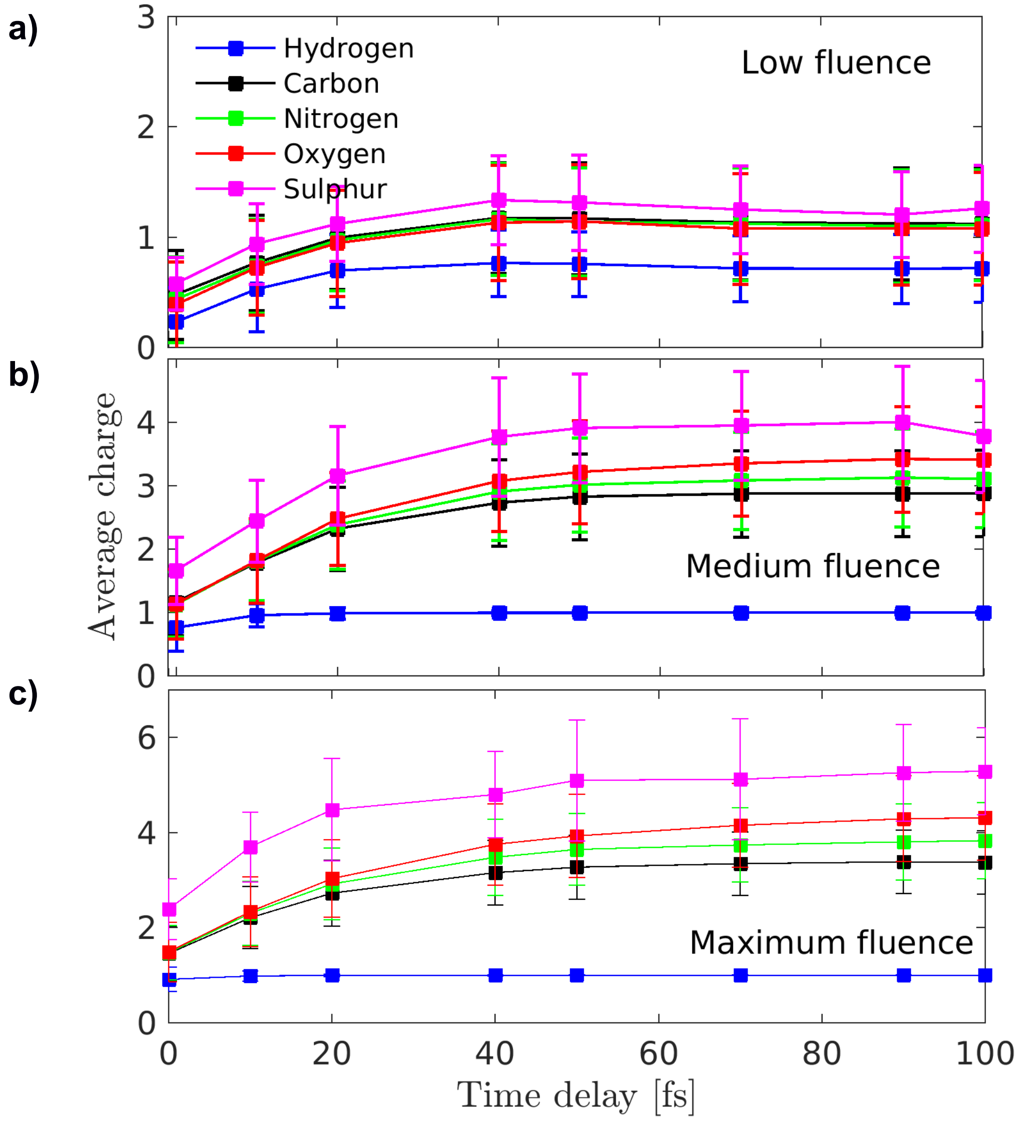

Supplementary Figure 15. **XMDYN results: Evolution of S charge.** Average charge as a function of time delay between pump and probe pulses. The blue, black, green, red and magenta curves represent the charge state for hydrogen, carbon, nitrogen, oxygen and sulphur atoms respectively. The pulse fluences considered are: (**a**) F_{low}= 8.8 x 10^{11} ph μm^{-2}, (**b**) F_{med} = 4.4 x 10^{12} ph μm^{-2}, and (**c**) F_{max} = 7.0 x 10^{12} ph μm^{-2}; the average charge was calculated, using 100 trajectories for each time delay. Due to the intense irradiation, the thaumatin sample becomes strongly ionized. Sulphur atoms, as the heaviest atomic constituents, reach the highest charge states, up to +5. The error bars are the standard deviation of the charge values derived from different trajectories.

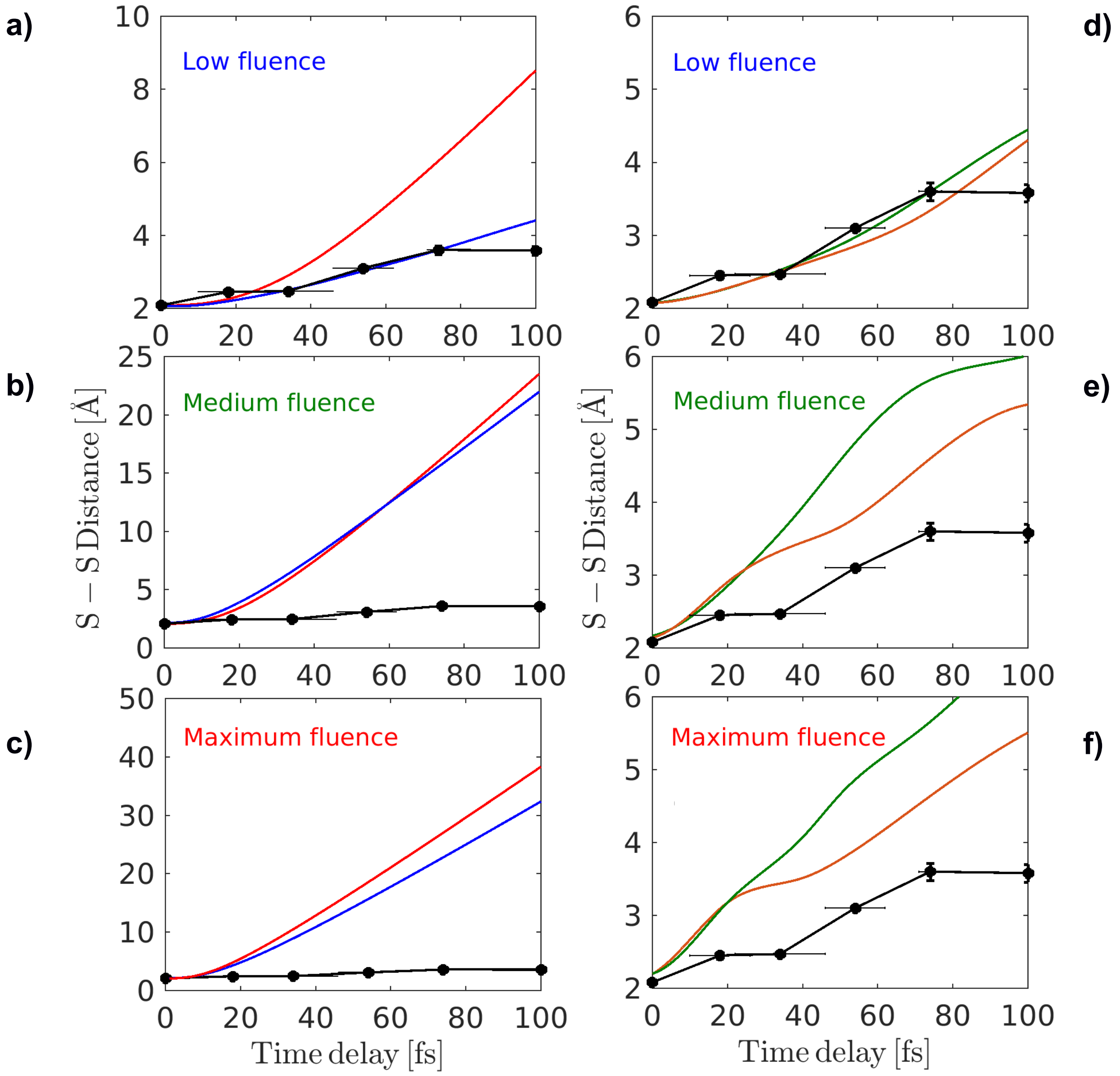

Supplementary Figure 16. XMDYN results: Comparison of various models. The S-S distances within the disulphide bridge obtained with different models as a function of pump-probe time delay and pump pulse fluence F, and compared to experimental results for thaumatin. Three fluence cases were studied: (**a**) F_{low} (8.8 x 10^{11} ph µm$^{-2)}$, (**b**) F_{med} (4.4 x 10^{12} ph µm^{-2}), and (**c**) F_{max} (7.0 x 10^{12} ph µm^{-2}). The black curve shows the experimental results. In plots (**a-c**), the red curves represent the evolution of an isolated S-S pair in vacuum after its irradiation with the corresponding X-ray pulse, and the blue curves show the S-S atom separation after assigning the time-dependent charge from Supplementary Fig. 15 to the S atoms of an isolated S-S pair. In plots (**d-f**), the evolution of the S-S distance within the thaumatin unit is shown: (i) when switching off the Coulomb interactions between S ions and free electron (brown curve), and (ii) when switching off the Coulomb interactions between S ions and non-S ions (green curve). The dissociation of the disulphide bridge is strongly influenced both by the high charge of its atomic constituents and by the charged environment of the bridge consisting of free electrons and non-S ions.

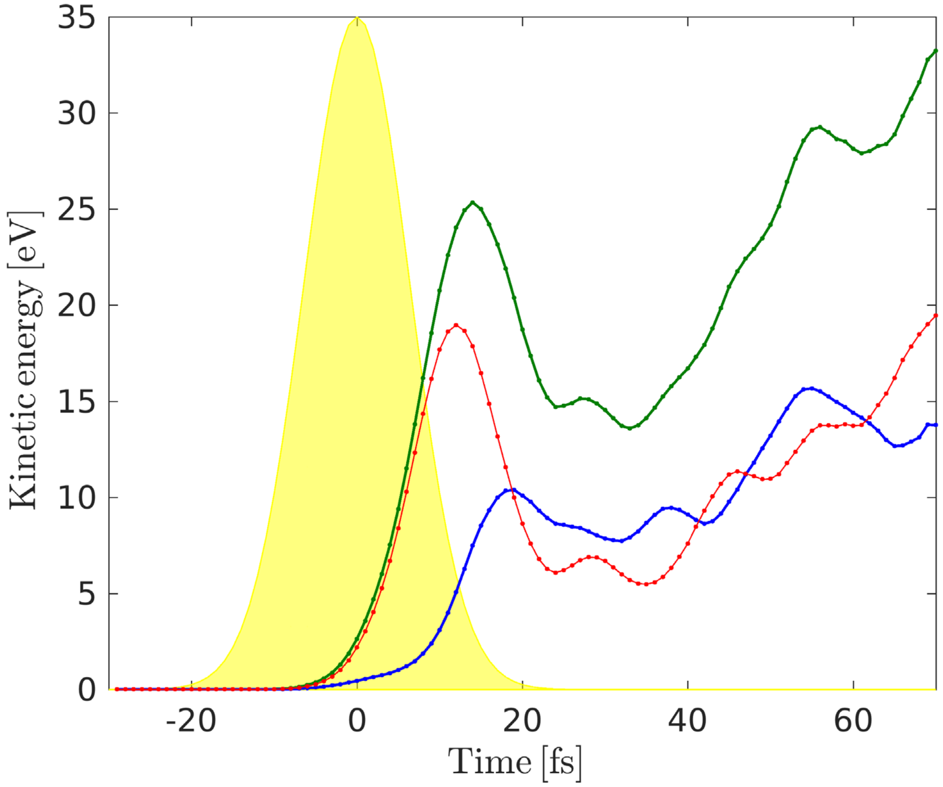

Supplementary Figure 17. XMDYN results: Average kinetic energy of S-S bridge Average kinetic energy of the S-S pair: The total kinetic energy (green curve), the translational energy (blue curve), and the internal energy (red curve) are shown. The yellow area represents the Gaussian temporal beam profile. The predictions were obtained for the maximum fluence case (F_{max} = 7.0 x 10^{12} ph $\mu m^{-2)}$. The environmental effect significantly slows down the S-S separation and induces complex motion of the bridge with both translational and vibrational modes.

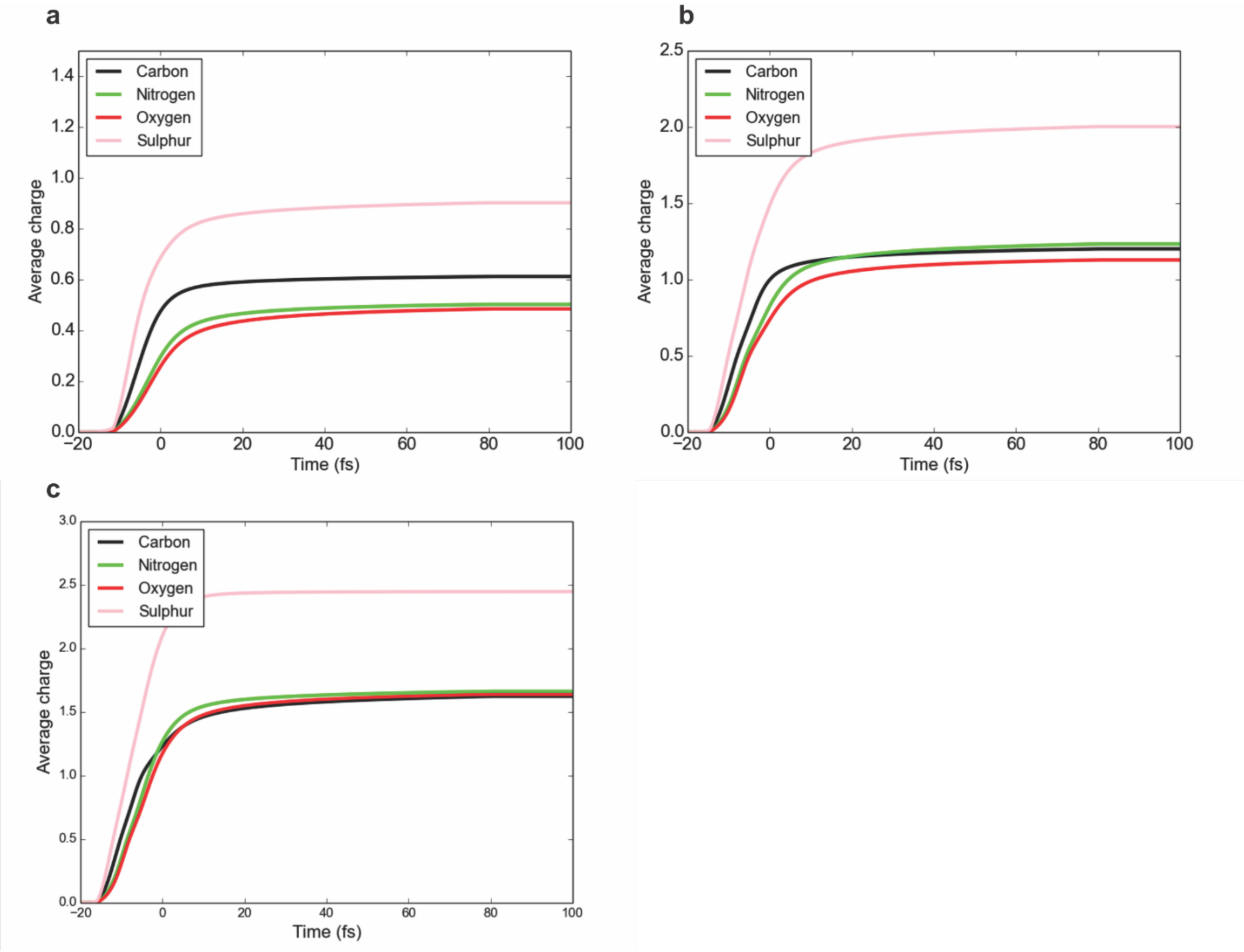

Supplementary Figure 18: **Charge states used in the hybrid plasma-MD model.** The same fluence cases were investigated as in the XMDYN analysis. (**a**) F_{low} (8.8 x 10^{11} ph μm^{-2}), (**b**) F_{med} (4.4 x 10^{12} ph μm^{-2}), and (**c**) F_{max} (7.0 x 10^{12} ph μm^{-2}). Note the temporal profile of the pulse is assumed to be Gaussian with a maximum located at 15fs in these plots.

Supplementary Table 1: Data and refinement statistics of Thaumatin. The same number of images were used for all datasets.

Time delay (nominal value)	Single pulse	18 fs (40fs)	35 fs (20fs)	54 fs (60fs)	76 fs (80fs)	100 fs (100 fs)
**Data collection**						
Space group	$P4_12_12$	$P4_12_12$	$P4_12_12$	$P4_12_12$	$P4_12_12$	$P4_12_12$
Cell dimensions (Å)						
a, *b*, *c* (Å)	57.8/ 57.8/ 150.3	57.8/ 57.8/ 150.3	57.8/ 57.8/ 150.3	57.8/ 57.8/ 150.3	57.8/ 57.8/ 150.3	57.8/ 57.8/ 150.3
α, β, γ (°)	90.0/ 90.0/ 90.0	90.0/ 90.0/ 90.0	90.0/ 90.0/ 90.0	90.0/ 90.0/ 90.0	90.0/ 90.0/ 90.0	90.0/ 90.0/ 90.0
Pulse energy (mJ)	0.30 ± 0.08	0.67 ± 0.14	0.93 ± 0.15	0.97 ± 0.14	0.95 ± 0.11	0.86 ± 0.14
No. indexed images	11000	11000	11000	11000	11000	11000
Wavelength (Å)	1.75	1.75	1.75	1.75	1.75	1.75
Resolution (Å)	22.9-2.3 (2.36-2.32)	22.9-2.3 (2.36-2.32)	22.9-2.3 (2.36-2.32)	22.9-2.3 (2.36-2.32)	22.9-2.3 (2.36-2.32)	22.9-2.3 (2.36-2.32)
$I / \sigma I$	7.4 (5.2)	7.7 (5.4)	8.0 (6.4)	8.6 (5.2)	7.6 (4.2)	6.1 (2.8)
R_{split} (%)	9.0 (22.2)	9.1 (23.1)	8.8 (14.5)	9.3 (21.2)	9.4 (31.2)	11.1 (54.6)
CC1/2	0.973 (0.929)	0.985 (0.911)	0.981 (0.965)	0.973 (0.926)	0.979 (0.871)	0.974 (0.695)
CC*	0.993 (0.981)	0.996 (0.976)	0.995 (0.991)	0.993 (0.980)	0.994 (0.965)	0.993 (0.905)
Completeness (%)	100 (100)	100 (100)	100 (100)	100 (100)	100 (100)	100 (100)
Multiplicity	139 (89)	137 (88)	167 (109)	148 (95)	146 (94)	128 (81)
Wilson B-factor (Å^2)	32.0	27.8	27.4	31.9	34.8	38.8
**Refinement**						
Resolution (Å)	23.0-2.3	23.0-2.3	23.0-2.3	23.0-2.3	23.0-2.3	23.0-2.3
No. reflections	11104	11104	11104	11104	11104	11104
R_{work} / R_{free}	0.138/0.170	0.168/0.212	0.140/0.170	0.163/0.203	0.144/0.176	0.147/0.188
No. atoms	1673	1673	1673	1673	1673	1673
Protein	1548	1548	1548	1548	1548	1548
S-atoms	16	16	16	16	16	16
Ligand	10 (TLA*)	10 (TLA)	10 (TLA)	10 (TLA)	10 (TLA)	10 (TLA)
Water	97	97	97	97	97	97
B-factors (Å^2)						
Protein	26.3	24.4	23.6	27.7	31.2	35.4
S-atoms	22.0	16.0	18.8	21.7	30.1	38.3
Ligand *	24.2 (TLA)	22.2 (TLA)	20.8 (TLA)	27.8 (TLA)	34.0 (TLA)	39.6 (TLA)
Water	29.5	27.6	28.0	32.6	38.9	43.5
R.m.s. deviations						
Bond lengths (Å)	0.016	0.021	0.016	0.015	0.013	0.012
Bond angles (°)	1.886	1.749	1.760	1.658	1.859	1.869
Ramachan. favored (%)	99.0	98.5	98.0	99.0	98.5	98.0
Ramachan. outliers (%)	0	0	0	0	0	0
MolProb. score (%)	1.73	2.05	2.05	2.20	2.10	2.08
**PDB code**	6SRJ	6SRQ	6SRK	6SRL	6SRO	6SRP

* L(+)-tartaric acid

The submitted coordinates represent the mean of 100 coordinate sets obtained by refining against 100 different integrated diffraction intensity datasets obtained by jackknife resampling of the diffraction snapshots. The coordinate header originates from one of the individual refinements. Cysteine residues were modeled as alanines and non-covalently attached sulphur atoms.

Supplementary Table 1: Data and refinement statistics of Lysozyme.Gd. The same number of images were used for all datasets.

Time delay (fs)<br>(nominal value)	Single pulse	35 fs<br>(20fs)	37 fs<br>(40fs)	62 fs<br>(60fs)	112 fs<br>(100 fs)	102 fs<br>(80 fs)
**Data collection**						
Space group	$P4_32_12$	$P4_32_12$	$P4_32_12$	$P4_32_12$	$P4_32_12$	$P4_32_12$
Cell dimensions (Å)						
a, *b*, *c* (Å)	79.0/ 79.0/ 39.5	79.0/ 79.0/ 39.5	79.0/ 79.0/ 39.5	79.0/ 79.0/ 39.5	79.0/ 79.0/ 39.5	79.0/ 79.0/ 39.5
α, β, γ (°)	90.0/ 90.0/ 90.0	90.0/ 90.0/ 90.0	90.0/ 90.0/ 90.0	90.0/ 90.0/ 90.0	90.0/ 90.0/ 90.0	90.0/ 90.0/ 90.0
Pulse energy (mJ)	0.33 ± 0.15	0.95 ± 0.18	0.99 ± 0.15	0.95 ± 0.14	0.93 ± 0.13	0.79 ± 0.15
No. indexed images	16000	16000	16000	16000	16000	11000
Wavelength (Å)	1.75	1.75	1.75	1.75	1.75	1.75
Resolution (Å)	22.8-2.3 (2.36-2.32)	22.8-2.3 (2.36-2.32)	22.8-2.3 (2.36-2.32)	22.8-2.3 (2.36-2.32)	22.8-2.3 (2.36-2.32)	22.8-2.3 (2.36-2.32)
$I / \sigma I$	9.5 (6.8)	12.0 (10.8)	12.4 (11.7)	12.4 (10.3)	10.6 (7.1)	8.5 (7.2)
R_{split}	8.5 (12.8)	8.4 (8.5)	8.3 (8.6)	7.8 (11.7)	9.1 (18.0)	10.7 (13.0)
CC1/2	0.983 (0.958)	0.980 (0.979)	0.981 (0.972)	0.984 (0.950)	0.978 (0.895)	0.977 (0.951)
CC*	0.995 (0.989)	0.995 (0.994)	0.995 (0.993)	0.996 (0.987)	0.994 (0.972)	0.994 (0.987)
Completeness (%)	100 (100)	100 (100)	100 (100)	100 (100)	100 (100)	100 (100)
Multiplicity	309 (215)	379 (265)	336 (233)	316 (218)	280 (188)	220 (151)
Wilson B-factor (Å^2)	37.9	27.5	28.9	34.2	39.1	36.3
**Refinement**						
Resolution (Å)	22.8-2.3	22.8-2.3	22.8-2.3	22.8-2.3	22.8-2.3	22.8-2.3
No. reflections	5315	5315	5315	5315	5315	5315
R_{work} / R_{free}	0.183/0.239	0.179/0.244	0.228/0.294	0.219/0.286	0.208/0.278	0.185/0.241
No. atoms	1080	1080	1080	1080	1080	1080
Protein	984	984	984	984	984	984
S-atoms	8	8	8	8	8	8
Ligand	58 ($GdDO_3$)<br>4 (NaCl)	58 ($GdDO_3$)<br>4 (NaCl)	58 ($GdDO_3$)<br>4 (NaCl)	58 ($GdDO_3$)<br>4 (NaCl)	58 ($GdDO_3$)<br>4 (NaCl)	58 ($GdDO_3$)<br>4 (NaCl)
Water	26	26	26	26	26	26
B-factors (Å^2)						
Protein	26.8	20.4	23.5	28.2	33.9	29.7
S-atoms	24.6	16.3	16.5	23.3	43.3	38.2
Ligand	46.2 ($GdDO_3$)<br>41.1 (NaCl)	39.9 ($GdDO_3$)<br>30.6 (NaCl)	42.6 ($GdDO_3$)<br>29.3 (NaCl)	45.5 ($GdDO_3$)<br>34.6 (NaCl)	49.1 ($GdDO_3$)<br>46.9 (NaCl)	47.4 ($GdDO_3$)<br>43.0 (NaCl)
Water	28.5	22.5	25.9	23.3	41.1	36.2
R.m.s. deviations						
Bond lengths (Å)	0.009	0.010	0.008	0.008	0.007	0.009
Bond angles (°)	1.683	1.658	1.51	1.611	1.680	1.786
Ramachan. favored (%)	96.8	97.6	95.2	96.0	96.0	96.0
Ramachan. outliers (%)	0	0	0	0	0	0
MolProb. score (%)	1.91	1.82	2.08	2.03	2.05	1.99
**PDB code**	6SR0	6SR1	6SR2	6SR3	6SR4	6SR5

The submitted coordinates represent the mean of 100 coordinate sets obtained by refining against 100 different integrated diffraction intensity datasets obtained by jackknife resampling of the diffraction snapshots. The coordinate header originates from one of the individual refinements. Cysteine residues were modeled as alanines and non-covalently attached sulphur atoms.

Supplementary Table 2: Data and refinement statistics of Thaumatin. All indexed images were used.

Time delay (nominal value)	Single pulse	18 fs (40fs)	35 fs (20fs)	54 fs (60fs)	76 fs (80fs)	100 fs (100 fs)
**Data collection**						
Space group	$P4_12_12$	$P4_12_12$	$P4_12_12$	$P4_12_12$	$P4_12_12$	$P4_12_12$
Cell dimensions (Å)						
a, *b*, *c* (Å)	57.8/ 57.8/ 150.3	57.8/ 57.8/ 150.3	57.8/ 57.8/ 150.3	57.8/ 57.8/ 150.3	57.8/ 57.8/ 150.3	57.8/ 57.8/ 150.3
α, β, γ (°)	90.0/ 90.0/ 90.0	90.0/ 90.0/ 90.0	90.0/ 90.0/ 90.0	90.0/ 90.0/ 90.0	90.0/ 90.0/ 90.0	90.0/ 90.0/ 90.0
Pulse energy (mJ)	0.30 ± 0.08	0.67 ± 0.14	0.93 ± 0.15	0.97 ± 0.14	0.95 ± 0.11	0.86 ± 0.14
No. indexed images	30000	15000	24000	14000	28000	11000
Wavelength	1.75	1.75	1.75	1.75	1.75	1.75
Resolution (Å)	22.9-2.3 (2.36-2.32)	22.9-2.3 (2.36-2.32)	22.9-2.3 (2.36-2.32)	22.9-2.3 (2.36-2.32)	22.9-2.3 (2.36-2.32)	22.9-2.3 (2.36-2.32)
$I / \sigma I$	11.9 (8.5)	9.0 (6.3)	11.6 (9.2)	9.6 (5.8)	11.9 (6.7)	6.1 (2.8)
R_{split} (%)	5.5 (13.5)	8.0 (19.8)	5.9 (10.6)	8.2 (20.5)	5.9 (20.5)	11.0 (55.3)
CC1/2	0.990 (0.973)	0.988 (0.925)	0.992 (0.981)	0.982 (0.933)	0.992 (0.937)	0.981 (0.670)
CC*	0.997 (0.993)	0.997 (0.980)	0.997 (0.995)	0.995 (0.982)	0.998 (0.983)	0.995 (0.895)
Completeness (%)	100 (100)	100 (100)	100 (100)	100 (100)	100 (100)	100 (100)
Multiplicity	378 (244)	186 (120)	364 (238)	188 (121)	372 (239)	128 (81)
Wilson B-factor (Å^2)	34.3	29.7	30.0	33.9	37.0	40.8
**Refinement**						
Resolution (Å)	23.0-2.3	23.0-2.3	23.0-2.3	23.0-2.3	23.0-2.3	23.0-2.3
No. reflections	11104	11104	11104	11104	11104	11104
R_{work} / R_{free}	0.136/0.172	0.173/0.215	0.140/0.177	0.163/0.205	0.140/0.177	0.144/0.187
No. atoms	1655	1655	1655	1655	1655	1655
Protein	1536	1536	1536	1536	1536	1536
S-atoms	16	16	16	16	16	16
Ligand	10 (TLA)	10 (TLA)	10 (TLA)	10 (TLA)	10 (TLA)	10 (TLA)
Water	79	79	79	79	79	79
B-factors (Å^2)						
Protein	28.6	26.5	25.9	29.8	33.4	37.8
S-atoms	23.9	17.4	20.6	23.8	31.8	40.0
Ligand	26.6 (TLA)	23.8 (TLA)	22.9 (TLA)	29.7 (TLA)	35.5 (TLA)	42.3 (TLA)
Water	30.4	28.6	28.8	33.6	39.0	43.5
R.m.s. deviations						
Bond lengths (Å)	0.015	0.017	0.017	0.018	0.016	0.014
Bond angles (°)	1.639	1.547	1.547	1.503	1.649	1.692
Ramachan. favored (%)	97.0	98.0	97.5	98.0	99.0	97.5
Ramachan. outliers (%)	0	0	0	0	0	0
MolProb. Clashscore (%)	1.80	1.79	1.91	1.81	1.86	1.94

Supplementary Table 2: Data and refinement statistics of Lysozyme.Gd All indexed images were used.

Time delay (fs) (nominal value)	Single pulse	35 fs (20fs)	37 fs (35fs)	62 fs (60fs)	112 fs (100 fs)	102 fs (80 fs)
**Data collection**						
Space group	$P4_32_12$	$P4_32_12$	$P4_32_12$	$P4_32_12$	$P4_32_12$	$P4_32_12$
Cell dimensions (Å)						
a, *b*, *c* (Å)	79.0/ 79.0/ 39.5	79.0/ 79.0/ 39.5	79.0/ 79.0/ 39.5	79.0/ 79.0/ 39.5	79.0/ 79.0/ 39.5	79.0/ 79.0/ 39.5
α, β, γ (°)	90.0/ 90.0/ 90.0	90.0/ 90.0/ 90.0	90.0/ 90.0/ 90.0	90.0/ 90.0/ 90.0	90.0/ 90.0/ 90.0	90.0/ 90.0/ 90.0
Pulse energy (mJ)	0.33 ± 0.15	0.95 ± 0.18	0.99 ± 0.15	0.95 ± 0.14	0.93 ± 0.13	0.79 ± 0.15
No. indexed images	26900	19400	16400	21600	17900	11600
Wavelength	1.75	1.75	1.75	1.75	1.75	1.75
Resolution (Å)	22.8-2.3 (2.36-2.32)	22.8-2.3 (2.36-2.32)	22.8-2.3 (2.36-2.32)	22.8-2.3 (2.36-2.32)	22.8-2.3 (2.36-2.32)	22.8-2.3 (2.36-2.32)
$I / \sigma I$	12.1 (8.7)	12.1 (11.9)	12.6 (11.8)	14.3 (11.9)	11.2 (7.6)	8.7 (7.3)
R_{split}	6.5 (9.5)	7.3 (7.5)	7.8 (8.9)	7.1 (9.4)	8.3 (16.1)	9.9 (12.9)
CC1/2	0.991 (0.977)	0.986 (0.985)	0.985 (0.974)	0.984 (0.972)	0.985 (0.910)	0.976 (0.952)
CC*	0.997(0.994)	0.996 (0.996)	0.996 (0.993)	0.996 (0.993)	0.996 (0.976)	0.994 (0.987)
Completeness (%)	100 (100)	100 (100)	100 (100)	100 (100)	100 (100)	100 (100)
Multiplicity	519 (360)	463 (325)	347 (241)	426 (294)	315 (212)	232 (159)
Wilson B-factor ($Å^2$)	38.0	27.5	29.0	34.1	39.1	36.3
**Refinement**						
Resolution (Å)	22.8-2.3	22.8-2.3	22.8-2.3	22.8-2.3	22.8-2.3	22.8-2.3
No. reflections	5315	5315	5315	5315	5315	5315
R_{work} / R_{free}	0.181/0.242	0.179/0.250	0.231/0.299	0.221/0.290	0.208/0.274	0.185/0.246
No. atoms	1074	1074	1074	1074	1074	1074
Protein	984	984	984	984	984	984
S-atoms	8	8	8	8	8	8
Ligand	58 ($GdDO_3$)<br>4 (NaCl)	58 ($GdDO_3$)<br>4 (NaCl)	58 ($GdDO_3$)<br>4 (NaCl)	58 ($GdDO_3$)<br>4 (NaCl)	58 ($GdDO_3$)<br>4 (NaCl)	58 ($GdDO_3$)<br>4 (NaCl)
Water	20	20	20	20	20	20
B-factors ($Å^2$)						
Protein	27.1	21.0	24.2	29.6	35.4	30.6
S-atoms	24.5	14.9	15.0	23.5	43.9	37.3
Ligand	45.9 ($GdDO_3$)<br>40.5 (NaCl)	41.0 ($GdDO_3$)<br>31.5 (NaCl)	40.9 ($GdDO_3$)<br>27.4 (NaCl)	44.3 ($GdDO_3$)<br>33.7 (NaCl)	48.6 ($GdDO_3$)<br>46.1 (NaCl)	46.8 ($GdDO_3$)<br>41.6 (NaCl)
Water	25.9	19.6	25.7	31.3	38.9	35.2
R.m.s. deviations						
Bond lengths (Å)	0.011	0.014	0.012	0.012	0.011	0.013
Bond angles (°)	1.540	1.521	1.283	1.398	1.546	1.638
Ramachan. favored (%)	96.8	96.8	96.8	96.8	96.0	96.0
Ramachan. outliers (%)	0	0	0	0	0	0
MolProb. score (%)	1.91	1.82	1.85	1.92	1.94	1.98

Supplementary Table 3: Analysis of disulphide bonds in lysozyme (PDB code 4ET8) and thaumatin (PDB code 3ZEJ) according to Wong and Hogg[4]. LH: left-handed, RH, right-handed, -LH Spiral, negative left-handed Spiral. See http://149.171.101.136/python/disulfideanalysis/ for details.

Protein	Cys1 residue	Cys1 secondary structure	Cys1 solvent accessibility [Å^2]	Cys2 residue	Cys2 secondary structure	Cys2 solvent accessibility [Å^2]	Strain energy kJ/mol	Cα distance of Cys1,2 [Å]	Classification
Lysozyme	30	α helix	0	115	H-bonded turn	0	9.12	6.05	-LH Spiral
Lysozyme	76	H-bonded turn	16	94	α helix	4	3.93	5.52	-RH Spiral
Lysozyme	64	loop/irreg.	0	80	3/10 helix	1	7.15	4.93	-/+RH Hook
Lysozyme	6	α helix	42	127	loop/irreg.	20	4.10	5.16	-LH Spiral
Thaumatin	71	loop/irreg.	1	77	loop/irreg.	0	6.77	5.72	+/-LH Spiral
Thaumatin	159	H-bonded turn	10	164	loop/irreg.	21	5.74	5.24	+/-RH Staple
Thaumatin	134	loop/irreg.	6	145	in isolated β-bridge	0	7.01	5.82	-RH Spiral
Thaumatin	9	bend	1	204	bend	5	10.29	4.91	-RH Hook
Thaumatin	56	β strand	6	66	β strand	8	15.53	3.91	-R/H Staple
Thaumatin	121	bend	33	193	β strand	1	8.47	5.27	-RH Hook
Thaumatin	126	β strand	1	177	loop/irreg.	4	6.54	5.30	-RH Hook
Thaumatin	149	α helix	23	158	α helix	30	7.59	4.80	-RH Hook
